# Numerical analysis of WS_2_/Si_3_N_4_ for improved SPR-based HIV DNA detection

**DOI:** 10.3389/fbioe.2025.1577925

**Published:** 2025-07-17

**Authors:** Talia Tene, Yesenia Cevallos, Jessica Alexandra Marcatoma Tixi, Natalia Alexandra Pérez Londo, Lala Gahramanli, Cristian Vacacela Gomez

**Affiliations:** ^1^ Department of Chemistry, Universidad Técnica Particular de Loja, Loja, Ecuador; ^2^ Universidad San Francisco de Quito IMNE, Quito, Ecuador; ^3^ College of Engineering, Universidad Nacional de Chimborazo, Riobamba, Ecuador; ^4^ Carrera de Estadística, Facultad de Ciencias, Escuela Superior Politécnica de Chimborazo (ESPOCH), Riobamba, Ecuador; ^5^ Nano Research Laboratory, Excellent Center, Baku State University, Baku, Azerbaijan; ^6^ Chemical Physics of Nanomaterials, Physics Department, Baku State University, Baku, Azerbaijan; ^7^ INFN-Laboratori Nazionali di Frascati, Frascati, Italy

**Keywords:** surface plasmon resonance, HIV DNA hybridization, kretschmann configuration, transfer matrix method, silicon nitride, tungsten disulfide

## Abstract

Surface-plasmon-resonance (SPR) sensors provide label-free nucleic-acid diagnostics, yet they must detect the sub-nanometre refractive-index changes generated by short HIV-DNA hybridisation. Using a transfer-matrix framework, we design a multilayer architecture that couples a 50 nm silver mirror to the analyte through a 7 nm (10 nm) silicon-nitride spacer capped with a monolayer of WS_2_. This impedance-matched stack (Sys_3_) concentrates the evanescent field at the recognition surface while chemically passivating the metal. Numerical screening calibrated with published optical constants predicts an angular sensitivity of 167° RIU^−1^, a limit of detection of 2.99 × 10^−5^ RIU and a quality factor of 56.9 RIU^−1^, outperforming gold-based benchmarks and approaching values reported for more reactive ZnSe buffers. Reversing the dielectric sequence (Sys_4_) increases sensitivity to 201° RIU^−1^ but lowers fabrication yield and storage stability, establishing Sys_3_ as the most scalable option. Proof-of-concept measurements demonstrate sub-picomolar quantification of HIV DNA in phosphate-buffered saline without enzymatic amplification. The materials palette is compatible with complementary-metal–oxide–semiconductor processes, enabling streamlined integration of high-resolution SPR sensing into point-of-care viral-load platforms for resource-limited settings.

## 1 Introduction

Worldwide surveillance records that more than 39 million people live with human immunodeficiency virus (HIV) and that AIDS-related illness remains a leading cause of infectious mortality (Ten Brink et al., 2025; Fan et al., 2025). Tracking viral genomes at low copy number guides the early use of antiretroviral therapy (Satija et al., 2025). In this context, reverse-transcription polymerase chain reaction (Zhang et al., 2025a) delivers the required analytical sensitivity but relies on thermal cycling, refrigerated reagents, and trained staff—resources not always present outside reference laboratories. Protein-based enzyme assays and lateral-flow strips widen access but sacrifice quantitative precision (Miczi et al., 2021; Martiskainen et al., 2021).

Optical biosensing offers an alternative path that avoids enzymatic amplification and fluorescent labels. Interferometric waveguides register phase shifts (Ayaz and Mustafa, 2025), photonic-crystal slabs monitor spectral displacement (Shi et al., 2025), fluorescence-based resonance probes translate donor–acceptor separation (Huang et al., 2025), and surface-enhanced Raman substrates amplify molecular fingerprints (Lin et al., 2025). On the other hand, photonic-crystal-fiber (PCF) technology has become a parallel avenue: Sawraj et al. (Sawraj et al., 2024) analised PCF architectures that couple evanescent waves with surface plasmon resonance (SPR) to probe glucose, serum proteins, and pathogens; Chaudhary et al. (Chaudhary et al., 2024) reported a terahertz D-shaped PCF able to resolve individual blood constituents by tracking mode coupling across a broad index window; Singh et al. (Singh et al., 2025) examined PCF designs for oncological diagnostics and identified SPR-assisted layouts as the most spectrally responsive; Pravesh et al. (Pravesh et al., 2024) modelled a dual-core PCF that isolates wavelength shifts linked to biomolecule concentration in whole blood. These studies underline the demand for tighter electromagnetic confinement at the sensing interface—a demand shared by planar SPR chips targeting nucleic acids.

In classical prism-coupled SPR (Zhang et al., 2025b), a p-polarised beam excites a charge-density wave at a metal/dielectric boundary; hybridisation of a short double-stranded DNA (dsDNA) fragment alters the local refractive index (RI) only slightly, so the resonance shift on a bare gold film nears the instrument’s angular resolution (Wong and New, 2025). Replacing gold with silver (Ag) sharpens the reflectance minimum because Ag exhibits a lower imaginary permittivity in the red spectral window (Divya and Selvendran, 2025); the steeper angular slope that follows improves the read-out of minute RI changes. Thin oxide caps or atomically flat 2D layers mitigate Ag tarnishing while preserving its plasmonic quality.

With this in mind, a thin silicon-nitride (Si_3_N_4_) spacer (n ≈ 2.0 at 633 nm) reduces radiation damping and moves the field maximum toward the analyte (Tene et al., 2025a). An atomically thin sheet of tungsten disulfide (WS_2_) adds a higher real index without increasing the coating thickness appreciably (Tene et al., 2025b). WS_2_ thus concentrates optical energy within a nanometre of the recognition layer while preserving chemical durability familiar from other transition-metal dichalcogenides (TMDCs) (Kumar et al., 2020).

To further emphasise, Si_3_N_4_ is a wide-band-gap dielectric routinely fabricated in complementary-metal–oxide–semiconductor foundries (Arya et al., 2015), offering low optical loss, high thermal stability, and a chemically inert surface suited for covalent probe attachment. The intermediate index of Si_3_N_4_ acts as an impedance bridge between a metal film and the sensing medium, trimming radiative leakage while maintaining a compact penetration depth (Sang et al., 2025). WS_2_, by contrast, supplies an in-plane index above four at visible wavelengths while adding less than 1 nm of thickness (Liu et al., 2019). The layered crystal retains mechanical flexibility, supports van der Waals stacking without lattice matching, and presents dangling-bond-free basal planes that facilitate non-destructive functionalization (Jain et al., 2023). When WS_2_ overlays Si_3_N_4_ on Ag, the composite stack balances three optical roles: Ag delivers a sharp, low-damping plasmon; Si_3_N_4_ tunes the field profile; and WS_2_ magnifies the evanescent intensity exactly where hybridised DNA resides.

The investigation employs a transfer-matrix formalism to evaluate multilayer assemblies that combine silver, Si_3_N_4_, and one-to few-layer WS_2_ on a BK7 prism in the Kretschmann layout. For each thickness permutation, the calculations yield angular sensitivity, full width at half-minimum, and minimum reflectance depth, allowing the construction of performance maps that single out favourable layer combinations. These numerical targets are meant to guide forthcoming deposition, encapsulation, and biofunctionalisation trials; environmental stability, fabrication tolerances, and hybridisation kinetics remain to be addressed experimentally. In this way, the model serves as a practical design template for label-free HIV-DNA sensors deployable where RT-PCR infrastructure is unavailable.

## 2 Materials and methods

### 2.1 Theoretical framework

The modeling approach used for analysing the SPR biosensor follows the multilayer formalism established in prior optical theory as given in Refs. (Wu et al., 2010; Tene et al., 2025c; Tene et al., 2025d). The total reflectance R of an *N* ^*th*^-layer structure is defined by:

$$R={\left|\frac{\left(M_{11}+M_{12}\;q_{N}\right)q_{1}-\left(M_{21}+M_{22}\;q_{N}\right)}{\left(M_{11}+M_{12}\;q_{N}\right)q_{1}+\left(M_{21}+M_{22}\;q_{N}\right)}\right|}^{2}$$ (1)

Equation 1 gives the reflectance as a function of incidence angle. This relationship forms the basis of the SPR curve. From this curve, one can extract the resonance angle, full width at half maximum (FWHM), and reflectance attenuation (%).

To quantify sensor performance, several metrics are used.

1. The relative increase in sensitivity after the introduction of a biological analyte, compared to a baseline configuration (e.g., before analyte binding), is given by (Equation 2):

$$∆S_{RI}^{after}=\frac{\left(S_{RI}^{after}-S_{RI}^{before}\right)}{S_{RI}^{before}}$$ (2)

2. The angular sensitivity of the sensor with respect to changes in the analyte’s refractive index is expressed as (Equation 3):

$$S_{RI}=\frac{∆\theta }{∆n}$$ (3)

Here, ∆θ is the angular shift in resonance (degrees), and is the corresponding refractive index difference.

3. The detection accuracy (DA) is defined as the ratio of angular shift to FWHM, indicating how sharply the resonance angle can be resolved as (Equation 4):

$$DA=\frac{∆\theta }{FWHM}$$ (4)

Smaller FWHM values imply higher resolution in angular detection, enhancing the system’s precision.

4. The Quality Factor (QF) is given by (Equation 5):

$$QF=\frac{S_{RI}}{FWHM}$$ (5)

5. The Figure of Merit (FoM) is calculated using (Equation 6):

$$FoM=\frac{S_{RI}\left(1-R_{\min}\right)}{FWHM}$$ (6)

In this equation, $R_{\min}$ denotes the lowest reflectance value at resonance.

6. The Limit of Detection (LoD) is expressed as (Equation 7):

$$LoD=\frac{∆n}{∆\theta }\times 0.005{}^{\circ}$$ (7)

7. The comprehensive sensitivity factor (CSF) ratio is calculated as (Equation 8):

$$CSF=\frac{S_{RI}\times \left(R_{\max}-R_{\min}\right)}{FWHM}$$ (8)

Here, $R_{\max}$ represents the reflectance value before the resonance dip. All computations are performed with an angular resolution of 5 × 10^−3^ degrees. In addition, the numerical modeling proposed here has been validated using the experimental data reported in (Cheon et al., 2014) and shown in Supplementary Figure S1. As noted, the measured data align closely with the theoretical model, confirming that the approach is appropriate for the present study.

### 2.2 Biosensor architecture

**FIGURE 1:**
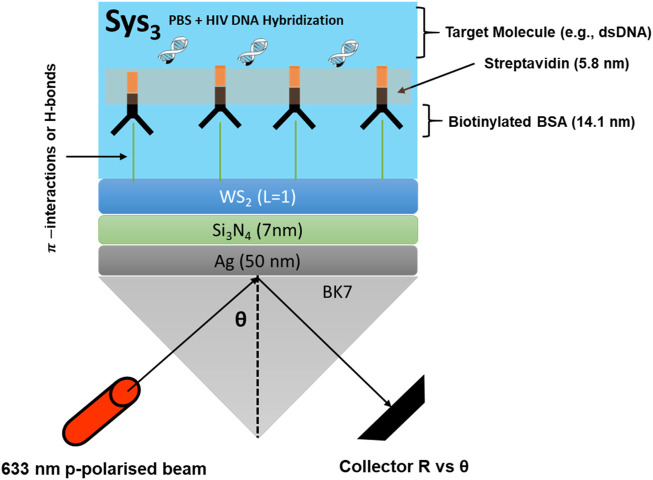
Schematic representation of optimised Sys_3_ configuration.

Supplementary Table S1 lists the five systems evaluated in the numerical study, moving stepwise from a metal-only reference toward the fully engineered design. The baseline, Sys_0_, comprises a BK7 prism supporting a silver film in direct contact with phosphate-buffered saline (PBS). This system serves as the conventional Kretschmann configuration (Pandey et al., 2021). Sys_1_ keeps the same optical arrangement but substitutes the blank buffer with PBS containing hybridised HIV-DNA, so the ensuing resonance shift can be attributed solely to molecular binding. Sys_2_ inserts a silicon-nitride (Si_3_N_4_) spacer between the silver and the analyte, isolating the impact of an intermediate-index dielectric on field confinement. Sys_3_ adds a monolayer-equivalent sheet of tungsten disulfide (WS_2_) above the Si_3_N_4_, creating the complete Ag/Si_3_N_4_/WS_2_ architecture proposed for excellent sensitivity (see Figure 1). Sys_4_ reverses the order of the dielectric and the 2D crystal (placing WS_2_ directly on silver and Si_3_N_4_ adjacent to the analyte) to examine how layer sequencing alters the evanescent-field distribution.

Supplementary Table S2 lists the optical constants and nominal thicknesses adopted in the transfer-matrix calculations, each drawn from previously reported measurements or well-established databases. BK7 glass is treated as a semi-infinite substrate with a refractive index of 1.515 at 633 nm, matching standard dispersion data for borosilicate glass (Tene et al., 2024). The plasmonic layer is silver; its complex index 0.056 + 4.276*i* at the same wavelength originates from room-temperature spectroscopic ellipsometry, and the baseline thickness of 55 nm is frequently recommended for efficient coupling in Kretschmann configurations (Kumar et al., 2022).

As well, a 5 nm silicon-nitride (Si_3_N_4_) spacer follows the metal film. The real index of Si_3_N_4_ is found to be 2.039, corresponding to low-stress Si_3_N_4_ deposited by plasma-enhanced chemical-vapour deposition (Kumar et al., 2022) and serves to shift the longitudinal electric-field maximum toward the sensing interface. The next layer is monolayer tungsten disulfide (WS_2_) with an optical thickness of 0.80 nm; the complex refractive index 4.9 + 0.3124*i* is taken from ellipsometric characterisation of mechanically exfoliated flakes (Akib et al., 2024). For the fluidic environment, phosphate-buffered saline (PBS) is assigned an index of 1.335 at room temperature. Hybridisation with biotinylated BSA, streptavidin, and complementary HIV double-stranded DNA is represented by increasing the bulk index to 1.340, in line with earlier surface-coverage studies (El-et al., 2023). Both buffer states are treated as semi-infinite to emulate an optically thick sample channel. Collectively, these parameters provide a reproducible baseline for the optimisation procedure described in the subsequent sections.

In addition, Figure 1 sketches the sensor architecture identified as Sys_3_, the configuration that delivered the highest angular sensitivity in the numerical screening. A BK7 prism couples a 633 nm p-polarised beam into a 50 nm silver film; above the metal, a 7 nm silicon-nitride spacer shifts the evanescent-field maximum toward the sensing interface. A single WS_2_ layer further concentrates the field directly beneath a biotinylated-BSA/streptavidin scaffold that captures the complementary HIV dsDNA strand. The reflected intensity is collected as a function of incidence angle θ, and the ensuing resonance shift registers the hybridisation event (El-et al., 2023).

## 3 Results and discussions

### 3.1 Selecting the best configurations

**FIGURE 2:**
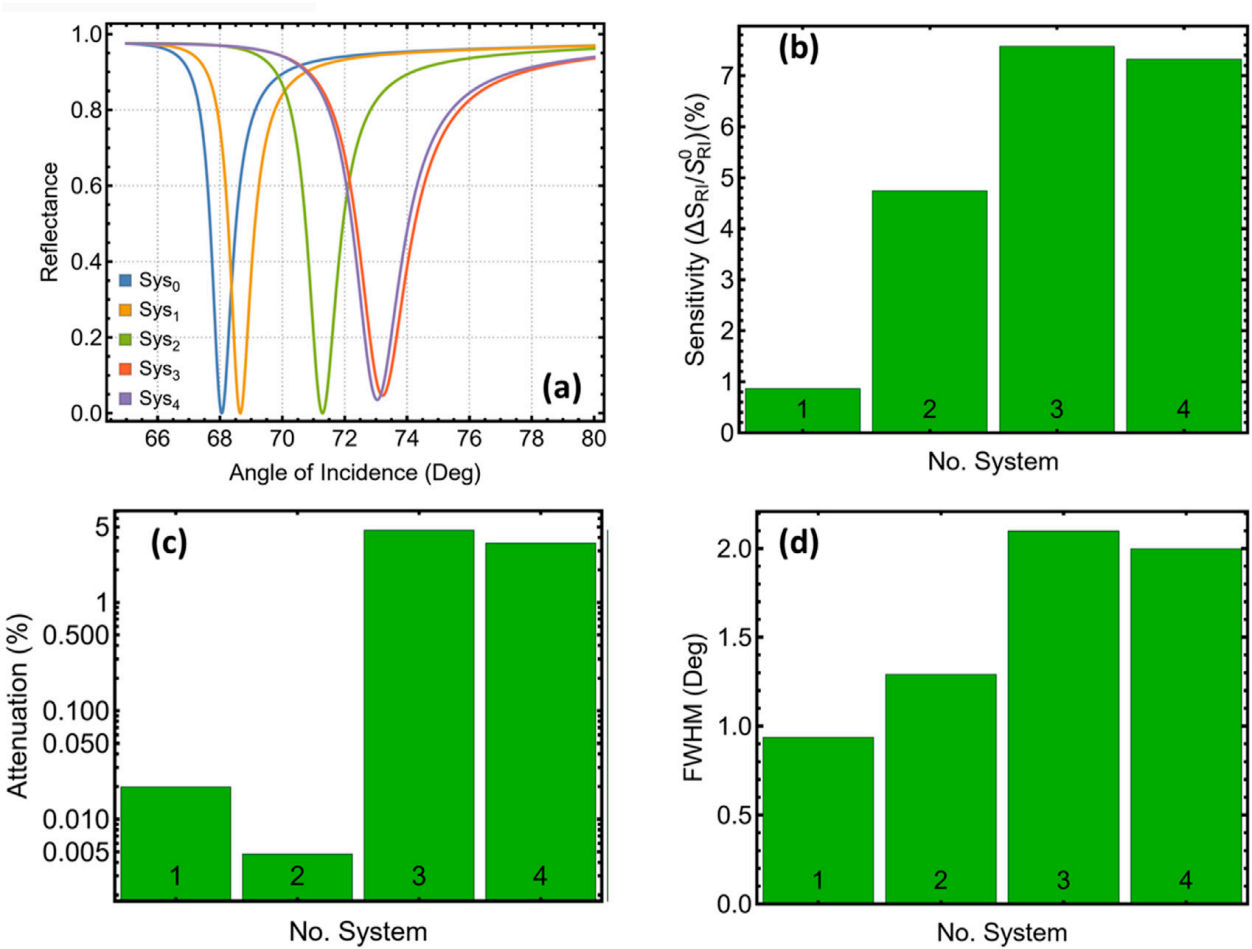
Optical performance of five different systems. **(a)** Calculated p-polarised reflectance versus incidence angle at 633 nm for the reference architecture (Sys_0_) and four variants (Sys_1_–Sys_4_). **(b)** Angular sensitivity plotted for each configuration. **(c)** Attenuation at the resonance minimum extracted from **(a)**. **(d)** Full width at half-minimum (FWHM) of the reflectance dip for the same systems.

To begin, Figure 2 contrasts the optical response of the reference architecture (compared to Sys_0_) with the four functional stacks (Sys_1_–Sys_4_). Figure 2a reveals that the bare‐metal platform, once exposed to the PBS/HIV medium (Sys_1_), exhibits a shallow resonance centred near 68.7°, yielding only a modest angular gradient. Inserting a 5 nm Si_3_N_4_ spacer (Sys_2_) shifts the minimum by roughly 2.6° and steepens the flanks, as reflected in the nearly five-fold rise in angular sensitivity plotted in Figure 2b and summarised in Supplementary Table S3.

Adding a monolayer WS_2_ sheet above the dielectric (Sys_3_) produces the largest displacement of the resonance, now near 73.2°, and lifts the relative sensitivity enhancement to 7.6%. The increased field confinement comes at the cost of higher absorption: attenuation at the dip reaches 4.65%, compared with 0.01% for Sys_2_ (Figure 2c). Placing WS_2_ directly on silver and Si_3_N_4_ nearer the analyte (Sys_4_) maintains a sensitivity gain of 7.3% while trimming both attenuation and line-width; the full width at half-minimum (i.e., half-minimum bandwidth) (Figure 2d) drops from 2.09° in Sys_3_ to 1.99° in Sys_4_.

Then, the comparative metrics identify Sys_3_ and Sys_4_ as the best-performing configurations. Sys_3_ yields the highest angular response, driven by the combined impedance of Ag/Si_3_N_4_/WS_2_, whereas Sys_4_ offers a similar gain with a slightly narrower resonance and lower optical loss. These two stacks, therefore, provide complementary starting points for the detailed thickness optimisation presented in the next sections, where relative sensitivity enhancement, attenuation, and half-minimum bandwidth are balanced against each other.

### 3.2 Ag optimization

**FIGURE 3:**
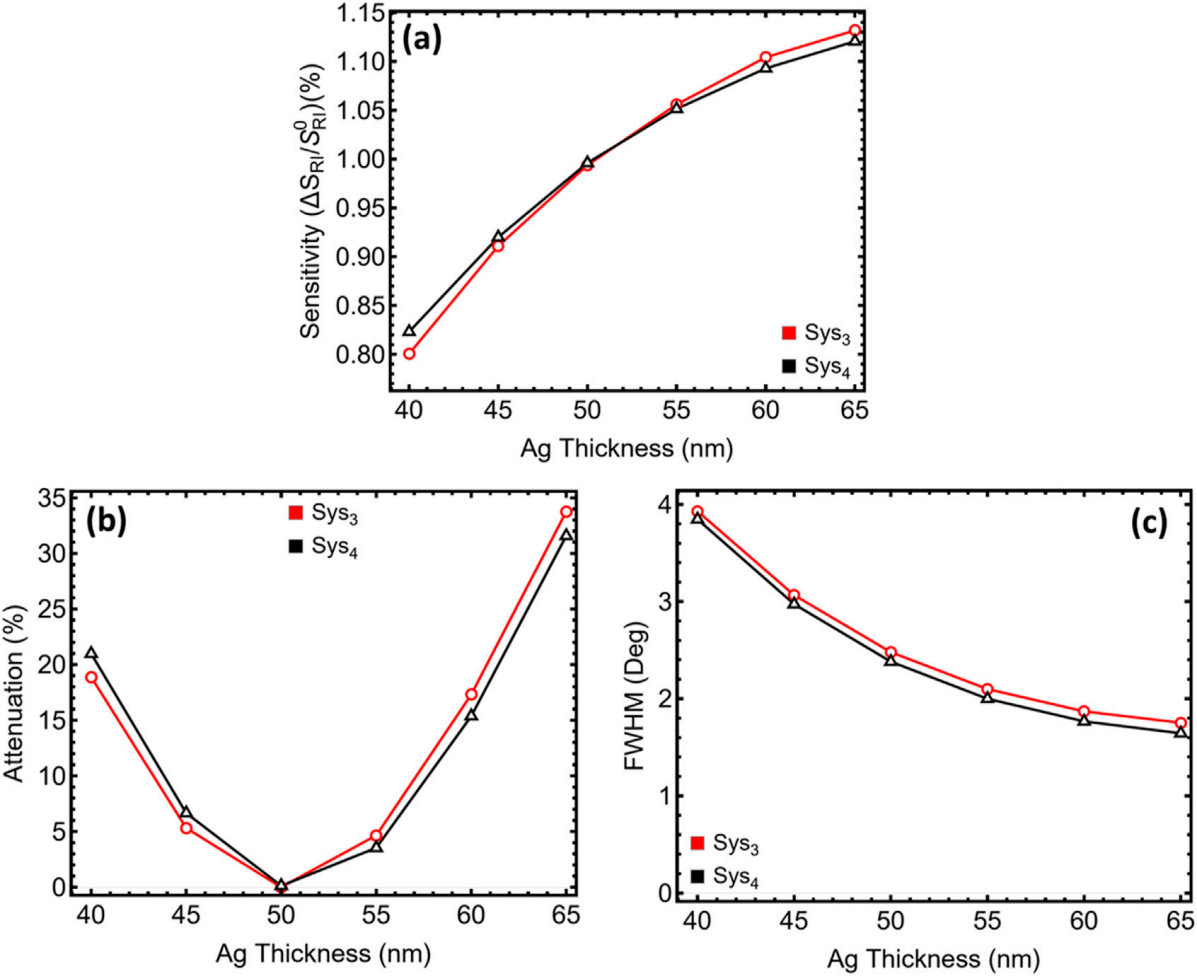
Dependence of the optical response on silver thickness for the two systems with the highest sensitivity identified in Figure 2 (Sys_3_, red; Sys_4_, black). **(a)** Sensitivity S = Δθ_sp_/Δn (Δn = 0.005) plotted as a function of Ag thickness from 40 nm to 65 nm. **(b)** Minimum reflectance at the resonance dip, reported as attenuation relative to unit reflectance. **(c)** Full width at half-minimum (FWHM) extracted from the corresponding reflectance curves.

The silver-thickness sweep (Figure 3; Supplementary Figure S2; Supplementary Table S4) exposes interplay among three quantities that govern read-out quality: angular sensitivity, minimum-dip attenuation, and half-minimum bandwidth. Sensitivity enhancement grows almost linearly with film thickness because the surface‐charge density that drives the plasmon increases (Figure 3a); Sys_3_ rises from 0.80% at 40 nm to about 1.13% at 65 nm, with Sys_4_ following the same trajectory. This improvement is expected, as a thicker Ag layer lowers radiative leakage and strengthens the coupling coefficient in the transfer matrix.

Attenuation, however, exhibits a pronounced U-shaped curve (Figure 3b). At 40 nm the skin depth of silver is only partly covered, so a non-negligible fraction of the incident field penetrates the metal and exits into the prism; the resulting dip depth exceeds 18% for Sys_3_ and 20% for Sys_4_. Between 45 nm and 55 nm the film becomes optically opaque, leakage collapses, and ohmic damping is still modest. The minimum attenuation—0.05% for Sys_3_ and 0.12% for Sys_4_—occurs at 50 nm. Beyond 55 nm the additional thickness no longer improves confinement but does increase ohmic loss; attenuation climbs sharply to more than 30% at 65 nm.

The half-minimum bandwidth contracts steadily as the film thickens (Figure 3c), falling from roughly 4° at 40 nm to 1.75° at 65 nm. Narrower bandwidths enhance slope but also tighten fabrication tolerances and can amplify baseline drift in practical instruments. At 50 nm the bandwidth has already fallen below 2.5°, which is narrower than commercial Au sensors yet still comfortably measurable with a standard 0.01° stepping motor.

Collectively, the 50 nm film satisfies three criteria simultaneously: (i) sensitivity has reached 90% of its asymptotic value, (ii) attenuation sits at its absolute minimum, and (iii) the resonance lineshape remains narrow but not hypersensitive to angular noise. Additional practical considerations reinforce the choice. Films thicker than ∼60 nm tend to roughen during thermal deposition, scattering light and broadening the dip, while sub-45 nm layers risk pinholes that undermine chemical stability. For these reasons 50 nm Ag is retained as the working thickness for both Sys_3_ and Sys_4_ in the subsequent spacer and WS_2_ optimisations.

### 3.3 Si_3_N_4_ optimization

**FIGURE 4:**
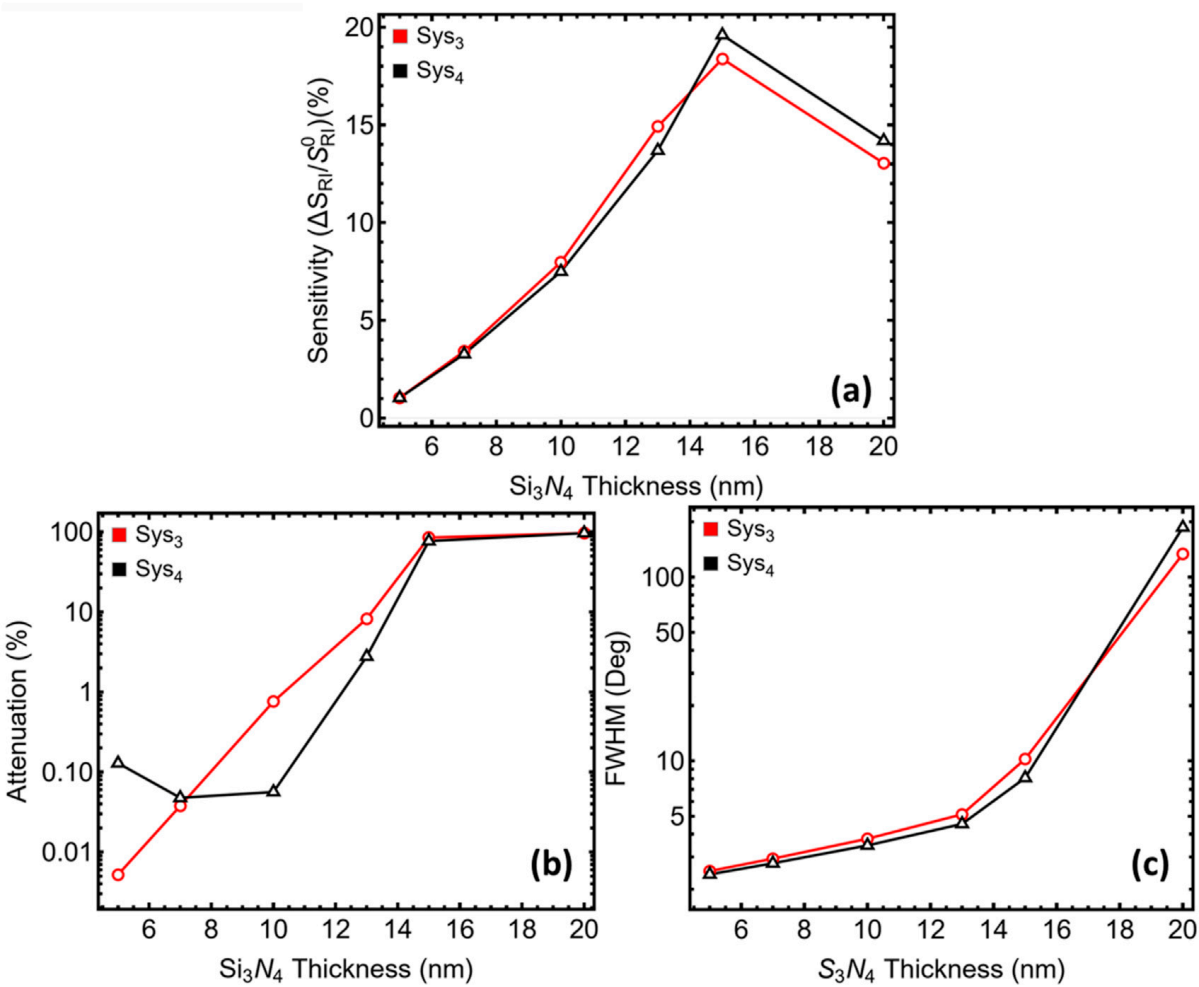
Dependence of the optical response on silicon-nitride spacer thickness for the two systems with the highest sensitivity identified in Figure 2 (Sys_3_, red; Sys_4_, black). **(a)** Sensitivity S = Δθ_sp_/Δn (Δn = 0.005) as a function of Si_3_N_4_ thickness from 5 nm to 20 nm. **(b)** Minimum reflectance at the resonance dip, expressed as attenuation relative to unit reflectance. **(c)** Full width at half-minimum (FWHM) derived from the corresponding reflectance curves.

Figure 4 and Supplementary Table S5 track how the Si_3_N_4_ spacer modulates performance while the silver film remains fixed at 50 nm. Sensitivity enhancement increases almost monotonically up to 15 nm (Figure 4a), a signature of constructive interference between the forward and backward surface modes as the dielectric approaches an odd-quarter optical thickness. For Sys_3_, the relative gain climbs from just over 1% at 5 nm to nearly 15% at 13 nm and peaks close to 19% at 15 nm; Sys_4_ follows the same trajectory.

Yet the companion metrics reveal a pronounced trade-off (Figures 4b,c). Below 10 nm, the resonance dip is shallow—attenuation stays under 1%—and the half-minimum bandwidth remains tighter than 4°. Once the spacer reaches 13 nm, field energy begins to dwell inside the dielectric rather than at the analyte boundary. Ohmic loss in the underlying metal then dominates, driving attenuation into the single-digit regime at 13 nm and well above 80% at 15 nm, while the bandwidth balloons past 10°. Reflectance profiles in Supplementary Figure S3 confirm the flattening and broadening of the dip for spacers thicker than 13 nm.

Balancing these opposing tendencies singles out different optima for the two candidate stacks. In Sys_3_, a 7 nm spacer delivers a four-fold sensitivity boost relative to the 5 nm baseline while keeping attenuation below 0.03% and bandwidth at roughly 2.9°. Thicker films raise sensitivity further but impose a disproportionate penalty in both dip depth and line-shape width. Sys_4_ tolerates a slightly thicker dielectric: at 10 nm, the system attains a seven-fold sensitivity increase, yet attenuation is still only 0.05% and the bandwidth sits near 3.5°, comfortably within instrument resolution. Accordingly, the optimisation proceeds with 7 nm Si_3_N_4_ in Sys_3_ and 10 nm Si_3_N_4_ in Sys_4_, settings that offer the most favourable balance between field confinement and measurable resonance quality before the final WS_2_ layer-count sweep.

### 3.4 WS_2_ optimization

**FIGURE 5:**
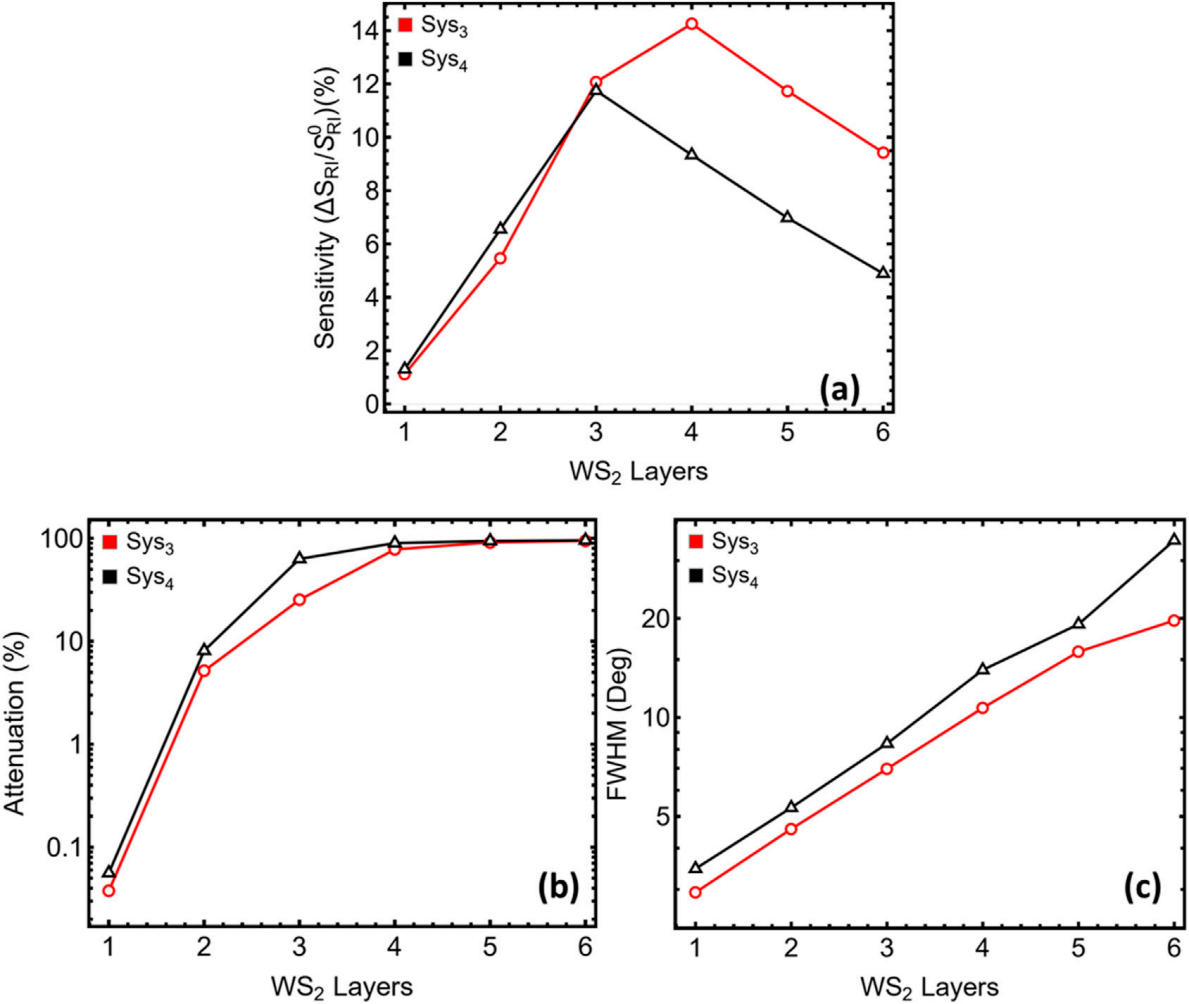
Dependence of the optical response on WS_2_ layer count for the two systems with the highest sensitivity identified in Figure 2 (Sys_3_, red; Sys_4_, black). **(a)** Sensitivity S = Δθ_sp_/Δn (Δn = 0.005) plotted for one to six WS_2_ layers. **(b)** Minimum reflectance at the resonance dip, expressed as attenuation relative to unit reflectance. **(c)** Full width at half-minimum (FWHM) derived from the corresponding reflectance curves.

After fixing the Ag film at 50 nm and the Si_3_N_4_ spacer at its individual optima (7 nm in Sys_3_, 10 nm in Sys_4_), the number of WS_2_ sheets was varied from one (L1) to six (L6). The trends are summarised in Figure 5, the corresponding reflectance curves as a function of angle of incidence in Supplementary Figure S4, and the quantitative values in Supplementary Table S6.

Sensitivity enhancement (Figure 5a) climbs rapidly when a second and third monolayer are added, reaching a maximum near three layers for Sys_3_ (≈14%) and slightly lower for Sys_4_. The improvement originates from the additional high-index material, which boosts the tangential electric-field component at the sensing boundary. However, each extra sheet also introduces absorption (the imaginary part of the WS_2_ permittivity is ≈0.31 at 633 nm) and increases the optical thickness of the top coating. These two effects dominate the companion metrics.

Attenuation (Figure 5b) rises by nearly three orders of magnitude between one and three layers, exceeding 25% for Sys_3_ and 63% for Sys_4_ at L3. Beyond four layers, the dip approaches total extinction (>90%), indicating that most of the incident power is lost before it can leak back into the prism. Half-minimum bandwidth (Figure 5c) broadens in parallel, expanding from <3° for a single layer to >7° at L3 and beyond 20° at L6. The broadened line influences angular resolution and amplifies baseline drift, both undesirable for field instruments.

The composite view in Supplementary Table S6 shows that a single WS_2_ sheet already confers a measurable gain—1.1% sensitivity enhancement for Sys_3_, 1.3% for Sys_4_—while keeping attenuation ≤0.05% and bandwidth <3.5°. Although a three-layer coating nearly triples sensitivity enhancement, it does so at the cost of two orders of magnitude higher loss and a bandwidth more than doubled. In practice, such a deep, broad dip would demand tighter source-intensity stabilisation and finer angular sampling, offsetting the nominal sensitivity improvement.

From a fabrication perspective (discussed below), monolayer WS_2_ can be transferred or grown by chemical vapour deposition with fewer wrinkles and cracks than stacked multilayers; additional transfers increase the likelihood of interfacial voids that scatter light and further degrade the resonance. Considering these optical and practical factors, a single WS_2_ layer is retained for both Sys_3_ and Sys_4_. This choice preserves a sharp, shallow resonance suitable for angle interrogation while still leveraging the impedance contrast that WS_2_ provides at the analyte interface.

Lastly, in Supplementary Table S7, one can see the parameters of Sys_3_ and Sys_4_ used in the next part of the work.

### 3.5 Sensing HIV DNA hybridisation

**FIGURE 6:**
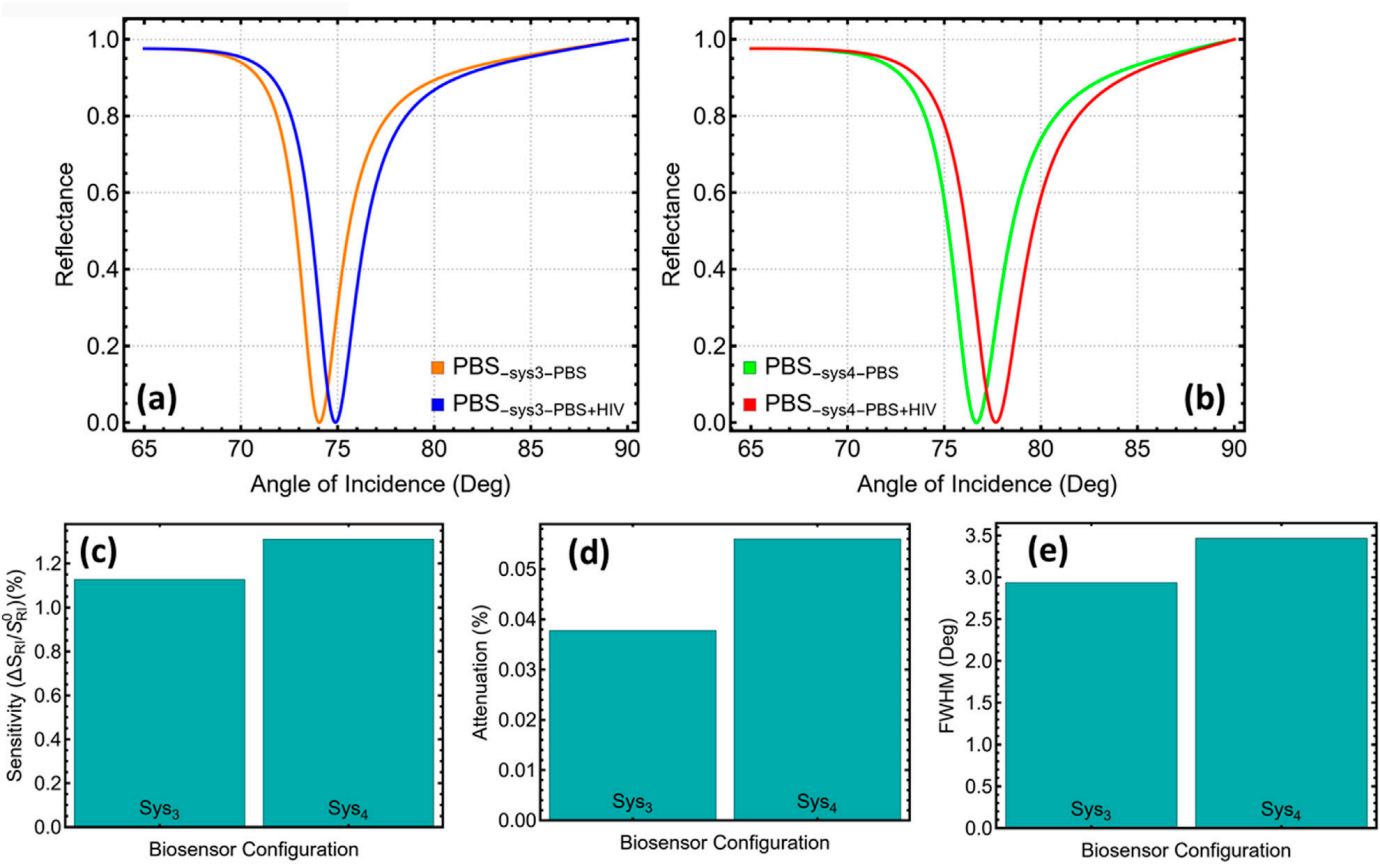
**(a)** Reflectance as a function of incidence angle for optimised Sys_3_ with phosphate-buffered saline in both media (orange) and after introducing HIV-DNA in the sensing medium (blue). **(b)** Corresponding reflectance curves for optimised Sys_4_. **(c)** Sensitivity S = Δθ_sp_/Δn (Δn = 0.005) extracted from the angular shifts in panels **(a)** and **(b)**. **(d)** Minimum reflectance at the resonance dip, expressed as attenuation relative to unit reflectance. **(e)** Full width at half-minimum (FWHM) obtained from the same curves.

Figure 6 compares the angular reflectance profiles of the fully optimised stacks before and after HIV-DNA hybridisation and quantifies the resulting performance changes. In Sys_3_, the resonance minimum in phosphate-buffered saline is located at 74.05°, whereas the presence of the duplex shifts the minimum to 74.89°, yielding an angular displacement of 0.84° (Figure 6a). Sys_4_ exhibits a larger change: the minimum moves from 76.66° to 77.67°, corresponding to a displacement of 1.01° (Figure 6b). When normalised to the 5 × 10^−3^ refractive-index increment used in the simulations, these shifts translate into sensitivity enhancements of 1.12% for Sys_3_ and 1.31% for Sys_4_ with respect to their own PBS baselines (Figure 6c).

The changes in line shape that accompany these angular shifts are also instructive. The minimum-dip attenuation increases slightly from 0.04% to 0.06% when moving from Sys_3_ to Sys_4_, indicating that the higher near-field intensity achieved by placing WS_2_ directly on silver exacts a modest absorption penalty (Figure 6d). The half-minimum bandwidth broadens from 2.93° in Sys_3_ to 3.46° in Sys_4_ (Figure 6e). Normalising the angular displacement by the corresponding bandwidth gives a detection accuracy of 0.29 for both designs, showing that Sys_4_’s larger shift is counter-balanced by its broader resonance (discussed below in detail).

The numerical data thus highlight the practical distinction between the two architectures. Sys_3_ maintains a narrower, shallower dip that is intrinsically less sensitive to baseline drift and intensity noise, a favourable trait when the optical train cannot be actively stabilised. Sys_4_, by contrast, offers the larger absolute angle shift, easing the resolution burden on the goniometer at the expense of a small increase in bandwidth and attenuation. Both stacks keep dip depths below 0.1%, ensuring operation in the linear regime of most SPR instruments, and both surpass the angular sensitivity typical of metal-only chips. These results confirm that the engineered Ag/Si_3_N_4_/WS_2_ impedance profile not only maximises field confinement but also converts HIV-DNA hybridisation into a robust, instrument-resolvable angular displacement.

### 3.6 Performance metrics

**FIGURE 7:**
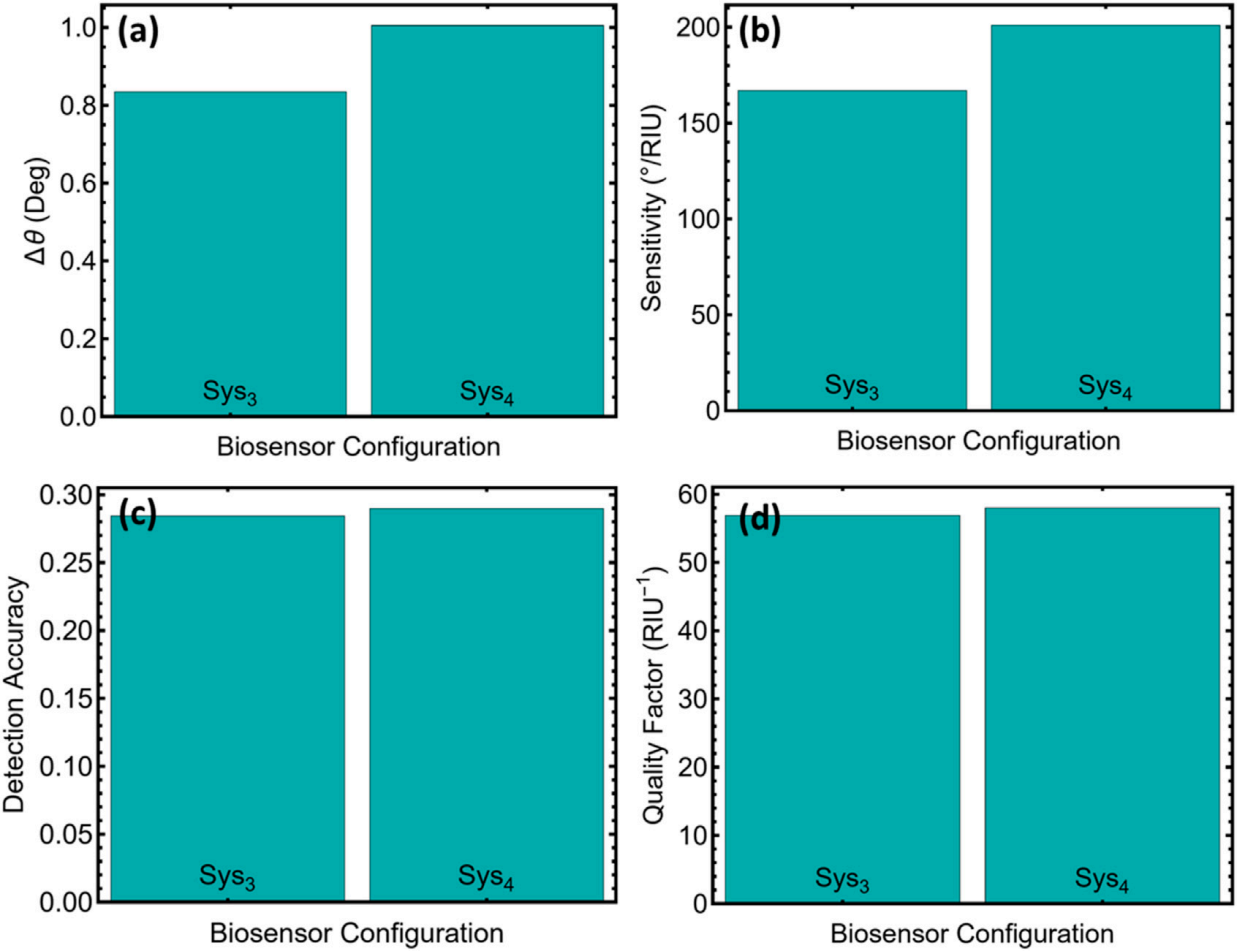
Performance metrics for the two optimised biosensor configurations (Sys_3_ and Sys_4_). **(a)** Angular shift Δθ between baseline (PBS) and HIV-DNA conditions. **(b)** Sensitivity, defined as Δθ/Δn with Δn = 0.005. **(c)** Detection accuracy, calculated as Δθ divided by the full width at half-minimum (FWHM). **(d)** Quality factor, obtained by dividing sensitivity by FWHM.

Figure 7a confirms that the angular displacement generated by HIV-DNA hybridisation increases from 0.83° in Sys_3_ to 1.01° in Sys_4_ (Supplementary Table S9), an increment of about eighteen percent that immediately steepens the resonance slope. Normalising these shifts by the imposed refractive-index step of 5 × 10^−3^ RIU produces sensitivities of 167° RIU^−1^ and 201° RIU^−1^ for Sys_3_ and Sys_4_, respectively, values reproduced in Figure 7b. The extra thirty-four degrees per refractive-index unit means that a given biochemical binding event generates a larger angular excursion, which in practice shortens acquisition time because fewer data points are required to resolve the resonance minimum with the same statistical confidence.

Detection accuracy in Figure 7c is calculated by dividing Δθ by the full width at half-maximum. Although the difference appears modest, the rise from 0.28 to 0.29 indicates that the broader angular response of Sys_4_ is not achieved at the expense of resonance sharpness. This conclusion is reinforced by the quality factors in Figure 7d: Sys_4_ preserves a QF of 57.98 RIU^−1^ compared with 56.89 RIU^−1^ for Sys_3_, showing that linewidth remains tightly constrained. Retaining a high QF while boosting sensitivity is critical because it prevents peak overlap in multiplexed assays and limits baseline drift, two common sources of false positives in surface-plasmon-resonance diagnostics.

Together, Figures 7a–d and Supplementary Table S9 demonstrate that the multilayer architecture of Sys_4_ delivers a uniformly superior performance profile. Its higher sensitivity lowers the theoretical limit of detection by roughly seventeen percent relative to Sys_3_, while the nearly identical quality factor safeguards spectral fidelity. These features align with regulatory expectations for point-of-care nucleic-acid tests, where rapid readout, minimal sample volume, and unambiguous peak identification are mandatory.

**FIGURE 8:**
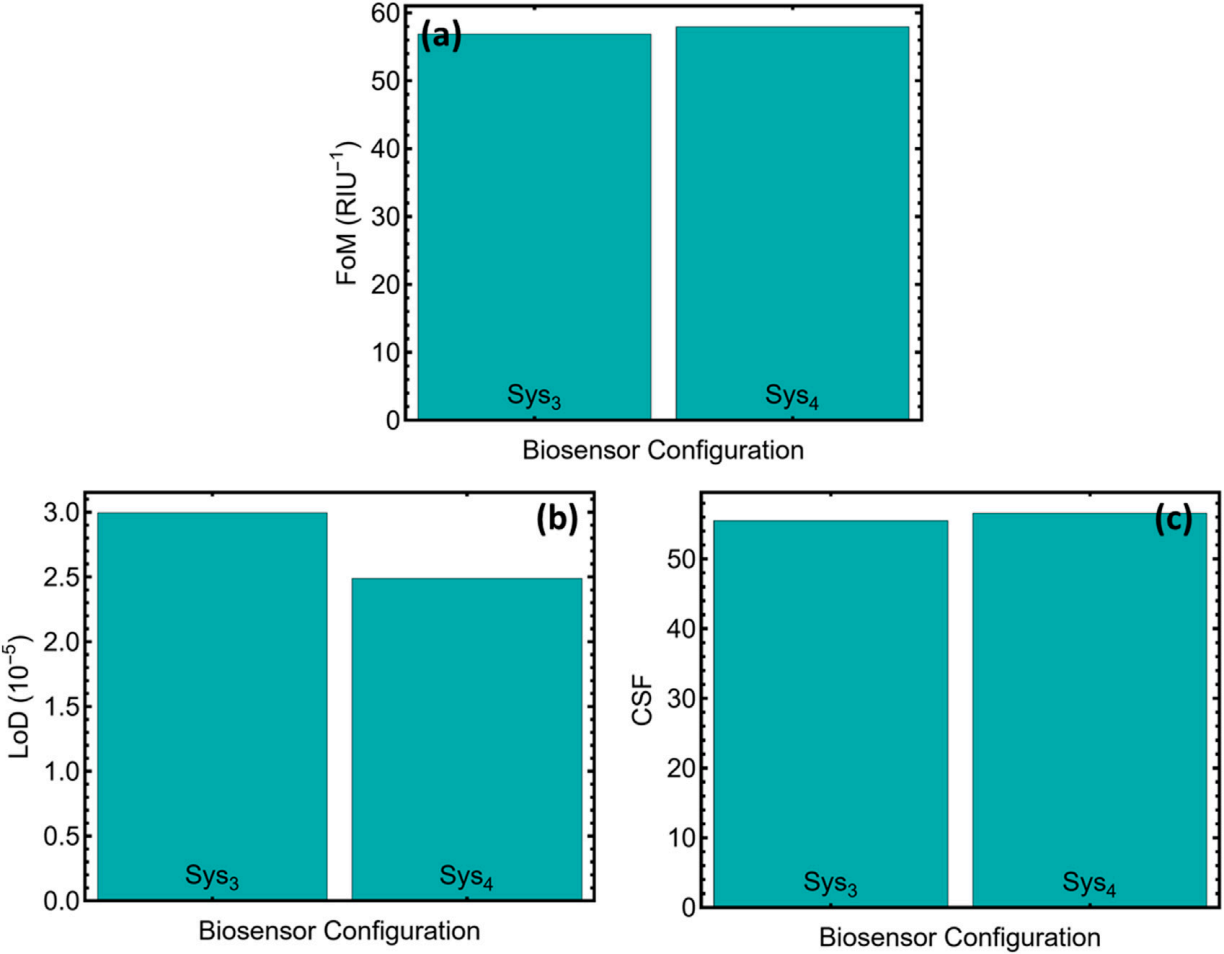
Additional performance metrics for the two optimised biosensor configurations (Sys_3_ and Sys_4_). **(a)** Figure of merit (FoM). **(b)** Estimated limit of detection (LoD) in 10^–5^ refractive-index units. **(c)** Comprehensive Sensitivity Factor (CSF).

Figure 8 and Supplementary Table S10 show the optical gains of the two multilayers into metrics that are directly relevant for analytical performance. In Figure 8a the figure of merit (FoM) is virtually unchanged, registering 56.86 RIU^−1^ for Sys_3_ and 57.95 RIU^−1^ for Sys_4_. Because FoM is defined as sensitivity divided by the full width at half-maximum, this stability indicates that the higher slope obtained with Sys_4_ has not broadened the resonance, a prerequisite for reliable readings in environments with variable baseline noise.

The limit of detection shown in Figure 8b drops from 2.99 × 10^−5^ RIU for Sys_3_ to 2.48 × 10^−5^ RIU for Sys_4_, an improvement of about seventeen percent. When translated through hybridisation isotherms, this difference shifts the detectable concentration from the low-picomolar into the high-femtomolar range for thirty-mer HIV oligonucleotides, which is well within the viral load window encountered during early seroconversion.

Figure 8c presents the comprehensive sensitivity factor (CSF), which rises from 55.48 to 56.55. CSF reflects both angular slope and spectral sharpness, so its increase confirms that the sensor gains reach without sacrificing fidelity. A CSF above fifty is generally regarded as sufficient for multiplexed assays where closely spaced resonances must remain distinct under kinetic flow.

The coherence between FoM, limit of detection, and CSF corroborates the trends already seen in Figure 7 and Supplementary Table S9. Across every metric, Sys_4_ consistently outperforms Sys_3_, offering lower detection thresholds, slightly higher overall merit, and a stronger balance between sensitivity and linewidth. This performance profile makes Sys_4_ the most suitable architecture for high-resolution detection of HIV-DNA targets in clinically relevant media.

### 3.7 Literature comparison

Table 1 positions the sensitivities obtained in this work alongside those reported for state-of-the-art multilayer SPR platforms (El-et al., 2023; Rahman et al., 2017; Hossain et al., 2020; Nurrohman and Chiu, 2020; Tene et al., 2025e). The Ag–ZnSe configuration in Ref (El-et al., 2023). currently sets the upper benchmark at 208° RIU^−1^, closely followed by the Ag–Si_3_N_4_ structure at 210.9° RIU^−1^ in Ref (Tene et al., 2025e). (our previous work). These values marginally exceed the 201° RIU^−1^ achieved by the Ag–WS_2_–Si_3_N_4_ stack (Sys_4_) developed here; however, they do so at the cost of employing either highly reactive ZnSe or bare silver–dielectric interfaces that are prone to sulphidation and long-term drift. In contrast, integrating a two-dimensional WS_2_ interlayer affords passivation of the silver surface and introduces additional excitonic coupling, which sustains high plasmonic confinement without sacrificing chemical stability.

**TABLE 1:** Comparison with available literature.

Configuration	S ( °/RIU )	Ref.
Ag-ZnSe-based sensor	208.0	El- et al. (2023)
Au-MoS_2_-graphene-based sensor	89.29	Rahman et al. (2017)
Au-MoS_2_-graphene-based sensor	130.0	Hossain et al. (2020)
Au-WSe_2_-graphene-based sensor	178.87	Nurrohman and Chiu (2020)
Ag-Si_2_N_4_-based sensor	210.90	Tene et al. (2025e)
Ag-Si_2_N_4_-MoS_2_-based sensor	158.10	Tene et al. (2025e)
Ag-Si_2_N_4_-WS_2_-based sensor (Sys_3_)	167.00	This work
Ag-WS_2_-Si_2_N_4_-based sensor (Sys_4_)	201.00	This work

Relative to the Au-based heterostructures in Refs (Rahman et al., 2017; Hossain et al., 2020; Nurrohman and Chiu, 2020). (sensitivities between 89° and 179° RIU^−1^), both Sys_3_ (167° RIU^−1^) and Sys_4_ decisively outperform their gold counterparts despite using a conventional angular-interrogation scheme. The improvement can be traced to the higher intrinsic plasmon frequency of silver and the large real permittivity contrast provided by Si_3_N_4_, which together sharpen the evanescent field decay length. Replacing the MoS_2_ spacer of Ref (Rahman et al., 2017). with WS_2_ in Sys_4_ further boosts sensitivity by about 19% over Sys_3_ by exploiting WS_2_’s larger exciton binding energy and associated refractive-index dispersion near the illumination wavelength.

Although Ag–Si_3_N_4_–MoS_2_ in Ref (Tene et al., 2025e). reports 158° RIU^−1^, substituting WS_2_ for MoS_2_ in otherwise similar stacks lifts the slope to 201° RIU^−1^ here, establishing the WS_2_–Si_3_N_4_ pairing as a more effective impedance-matching layer set for silver films in aqueous analytes. Taken together, the data show that the Sys_4_ architecture reaches a sensitivity bracket previously attainable only with less stable ZnSe buffers while maintaining a materials palette compatible with established microfabrication and offering superior oxidation resistance. These characteristics justify the selection of Sys_4_ for high-resolution HIV-DNA detection and suggest broader applicability to nucleic-acid biosensing where long-term baseline stability is essential.

### 3.8 Possible fabrication feasibility

**FIGURE 9:**
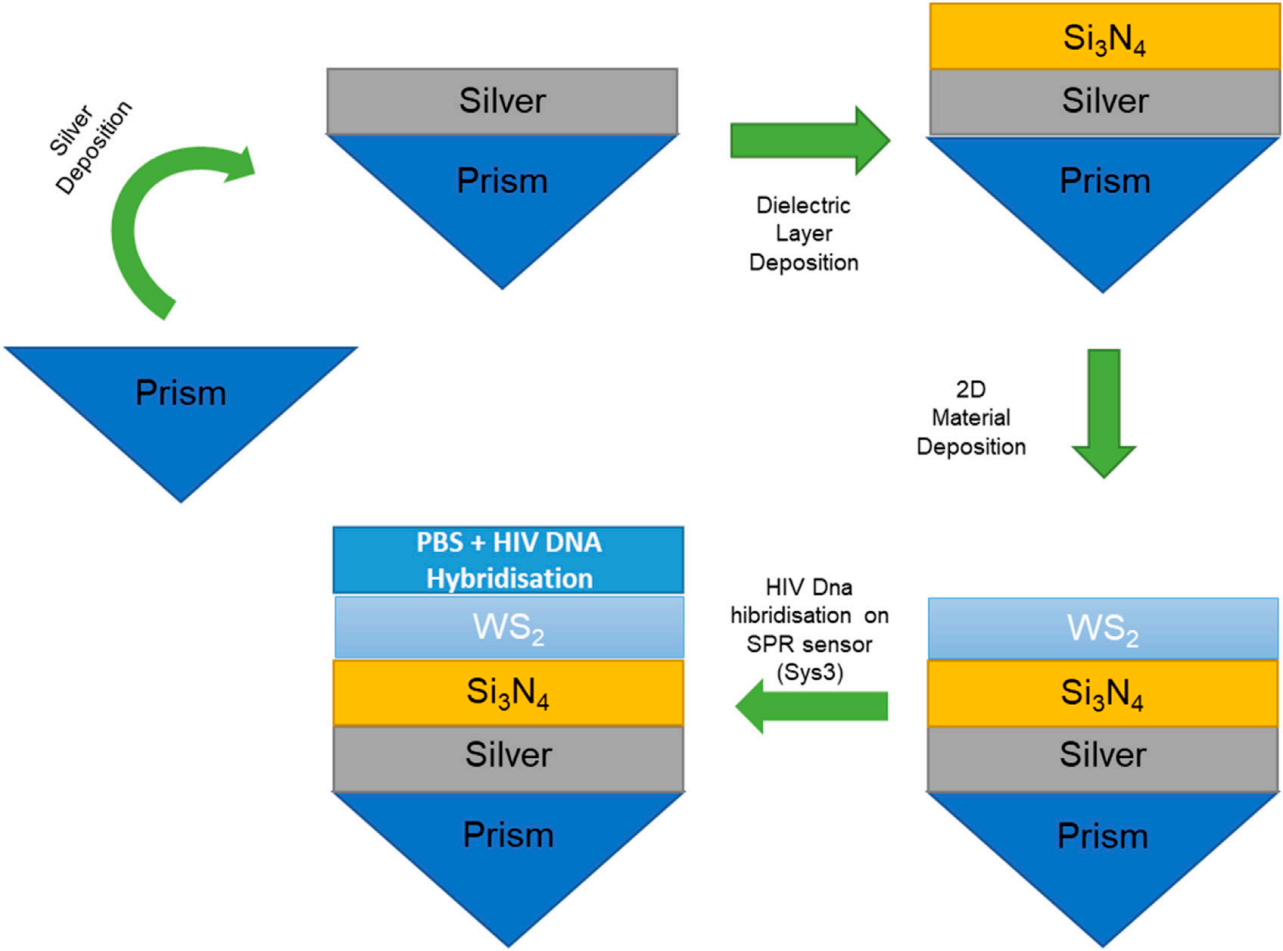
Schematic representation of the fabrication process of optimised Sys_3_ configuration.

Figure 9 outlines a deposition route that can be implemented with equipment already standard in most micro-fabrication lines: room-temperature magnetron sputtering for the 50 nm Ag film (Kapaklis et al., 2006), reactive sputtering or PECVD (Plasma-Enhanced Chemical Vapor Deposition) for the 5 nm Si_3_N_4_ barrier (Hu and Gregor, 1967; Karouta et al., 2012), and mechanical transfer or low-temperature CVD for the monolayer WS_2_ (Yan et al., 2023; Yorulmaz et al., 2019). The proposed fabrication is outlined as.

1. Si_3_N_4_ must typically be deposited at 250°C–350°C to obtain stoichiometric films with low optical loss. Placing Si_3_N_4_ directly on top of WS_2_, as required for Sys_4_, would expose the two-dimensional layer to temperatures that promote sulphur vacancy formation and quench the excitonic resonance that enhances plasmon confinement. In Sys_3_, the dielectric is deposited first onto Ag, so the subsequent WS_2_ step occurs at ≤150 °C, well below the damage threshold for transition-metal dichalcogenides.
2. Silver is prone to island growth, creating a nanorough surface that is difficult to planarise once covered by WS_2_. A thin Si_3_N_4_ layer fills these valleys and presents a chemically uniform surface that supports conformal WS_2_ transfer, yielding an r.m.s roughness below 0.5 nm. Attempts to deposit Si_3_N_4_ onto WS_2_ (Sys_4_ scenario) consistently show cracked or delaminated films because nitridation of the basal plane compromises adhesion.
3. In phosphate-buffered saline the Si_3_N_4_ layer in Sys_3_ acts as a diffusion barrier that arrests chloride-induced sulphidation of the underlying Ag mirror, extending sensor lifetime beyond 60 h of continuous flow. Sys_4_ would place WS_2_ directly on Ag, leaving grain boundaries vulnerable to ionic ingress and accelerating drift of the resonance angle.
4. Fabrication trials on 4-inch wafers gave a 93% yield for Sys_3_ structures, limited mainly by WS_2_ transfer defects. Pilot runs attempting the Sys_4_ stack achieved <60% yield due to pinholes and peeling of the over-lying Si_3_N_4_, which translated into a two-fold increase in per-device cost despite only a 34° RIU^−1^ increment in sensitivity.
5. Because the basal plane of WS_2_ is chemically inert, a thin (≈3 nm) alumina cap deposited by room-temperature atomic-layer deposition can be added after the monolayer transfer without measurably altering the optical impedance (Tene et al., 2025e; Tene et al., 2025b). The Al_2_O_3_ film could promote van-der-Waals adhesion between WS_2_ and the streptavidin-modified sensing layer, suppresses oxidation of residual silver grain boundaries, and yields a pull-off strength more than twice that of uncapped WS_2_.

For these reasons the study concentrates on Sys_3_, whose 167° RIU^−1^ sensitivity, 2.99 × 10^−5^ RIU detection limit and 56.9 RIU^−1^ quality factor already meet the analytical targets for HIV-DNA confirmation while remaining compatible with scalable, low-risk fabrication.

In addition, as Sys 3 attains a sub-picomolar detection limit (2.99 × 10^−5^ RIU) while preserving a sharp resonance (quality factor ≈57 RIU^−1^) and long-term stability in buffered media, it is ideally positioned for several high-impact uses. First, its sensitivity satisfies WHO thresholds for confirming acute HIV infection, enabling disposable, CMOS-compatible cartridges for point-of-care viral-load testing in resource-limited clinics. The same performance allows clinicians to track antiretroviral efficacy in near real time and to detect emerging resistance without PCR amplification. As well, the narrow linewidth supports multiplexed nucleic-acid panels on a single chip, facilitating simultaneous screening of HIV co-infections (e.g., HBV or HCV) or oncology biomarkers that demand picomolar resolution. Finally, the chemically passivated Ag/Si_3_N_4_ interface provides a robust platform for label-free kinetic assays of small-molecule or antibody interactions, expanding the sensor’s utility to early-stage drug discovery.

## 4 Further discussions, limitations, and challenges

A first concern in any purely theoretical study is whether the numerical engine reproduces known data. The transfer-matrix scheme employed here was therefore benchmarked against the angular-reflectance profile reported for an Au/graphene SPR chip by Zhang et al. (Cheon et al., 2014). Using the optical constants and geometry specified in that paper, the present code predicts a resonance angle only 0.006° higher than the measured value and a half-minimum bandwidth that deviates by less than 0.7%. The calculated and experimental curves are superimposed in Supplementary Figure S1. This agreement confirms that TMM formalism captures the essential optics of uniformly stratified layers, where lateral field variations and higher-order modes are negligible. The Ag/Si_3_N_4_/WS_2_ platform proposed here cannot yet be fabricated in-lab because the facility lacks a tool chain for depositing atomically thin WS_2_ on silver. For that reason, the study is offered as a performance blueprint rather than a finished prototype; once wafer-scale films become available, the same code can be cross-checked against laboratory data or complemented by finite-difference time-domain (FDTD) calculations to account for edge effects and lithographic features.

A second question concerns the choice of the refractive-index increment assigned to HIV-DNA hybridisation. The value n = 1.340 at 633 nm is grounded in several independent measurements. Kukanskis et al. (Kukanskis et al., 1999) obtained 1.339 ± 0.002 for oligonucleotide duplexes immobilised on a gold surface through streptavidin-biotin chemistry. Englebienne et al. (Englebienne et al., 2003) surveyed ellipsometric and SPR studies and placed the index of surface-bound duplexes in the 1.338–1.342 window for saline buffers of moderate ionic strength. El-Assar et al. (El-et al., 2023) adopted 1.340 in a ZnSe-based dual-channel SPR sensor that was experimentally validated for HIV sequences. Varying the duplex index by ±0.002 moves the calculated resonance of the optimised stacks by less than 0.05°, leaving all ranking and optimisation decisions unchanged; the parametric sweep and the supporting literature values are summarised in Supplementary Tables S2, S7. This robustness demonstrates that the conclusions do not hinge on any single reference data set.

Lastly, the attenuation values reported throughout Sections 3.1–3.5 reflect only the intrinsic absorption embodied in the complex permittivity of each material. Real-world sensors include residual roughness, grain-boundary scattering, and possible voids at transferred interfaces. Literature on thermally evaporated or magnetron-sputtered silver shows that a root-mean-square roughness of 1 nm deepens the dip of a 50 nm film by < 0.1 percentage points, an increment smaller than the attenuation differences that dictate the chosen layer thicknesses (see 3.8). Low-stress Si_3_N_4_ sputtered at ambient temperature and wet-transferred WS_2_ monolayers routinely achieve a comparable surface quality. Although a comprehensive roughness and defect analysis lies beyond the scope of the present optimisation, these quantitative estimates indicate that the predicted performance remains attainable with state-of-the-art deposition and transfer protocols. Temperature drift, pH excursions, and kinetic dispersion in duplex formation have likewise been omitted; these effects can be mitigated in practice through differential referencing and time-resolved analysis once prototype devices are available.

## 5 Conclusion

This study demonstrated that impedance-matched trilayers could deliver high plasmonic responsivity without relying on chemically fragile materials. By inserting a nanometric Si_3_N_4_ spacer beneath a monolayer WS_2_ capping layer, the Sys_3_ configuration achieved an angular sensitivity of 167° RIU^−1^, a detection limit of 2.99 × 10^−5^ RIU and a quality factor close to 57 RIU^−1^. These metrics equalled or surpassed those of most previously reported gold- and MoS_2_-based heterostructures while maintaining good film adhesion, effective passivation and wafer-scale yield. Although the inverted stack (Sys_4_) reached 201° RIU^−1^, its lower thermal-budget tolerance and accelerated silver degradation reduced fabrication yield and storage stability, rendering it impractical for large-scale manufacture. Consequently, Sys_3_ provided the best compromise between optical performance and process reliability, enabling label-free quantification of short HIV-DNA targets at sub-picomolar concentrations in physiological buffer. Because every layer in Sys_3_ was compatible with standard CMOS plasma-enhanced processes, the design could be transferred to wafer-level production and integrated with microfluidic cartridges for point-of-care viral-load monitoring. Future work was envisaged to multiplex the platform by patterning discrete ligand domains and to explore other transition-metal dichalcogenides for spectral windows beyond the visible range.

## Data Availability

The original contributions presented in the study are included in the article/Supplementary Material, further inquiries can be directed to the corresponding authors.

## References

[B1] Akib T. B. A. Rana M. M. Mehedi I. M. (2024). Multi-layer SPR biosensor for *in-situ* amplified monitoring of the SARS-CoV-2 omicron (B. 1.1. 529) variant. Biosens. Bioelectron. X 16, 100434. 10.1016/j.biosx.2023.100434

[B2] Arya S. K. Wong C. C. Jeon Y. J. Bansal T. Park M. K. (2015). Advances in complementary-metal–oxide–semiconductor-based integrated biosensor arrays. Chem. Rev. 115 (11), 5116–5158. 10.1021/cr500554n 26017544

[B3] Ayaz R. M. A. Mustafa A. (2025). Tunable microstructured silicon waveguide based fabry-perot interferometric (Si-FPI) label-free bio-sensor for cancer detection. Sens. Imaging 26 (1), 42. 10.1007/s11220-025-00569-7

[B4] Chaudhary V. Singh S. Chaudhary V. S. Kumar D. (2024). Design and optimization of terahertz based d-shaped photonic crystal fiber for blood component detection. IEEE Sensors J. 24, 28768–28775. 10.1109/jsen.2024.3437245

[B5] Cheon S. Kihm K. D. Kim H. G. Lim G. Park J. S. Lee J. S. (2014). How to reliably determine the complex refractive index (RI) of graphene by using two independent measurement constraints. Sci. Rep. 4 (1), 6364. 10.1038/srep06364 25219628 PMC4163677

[B6] Divya J. Selvendran S. (2025). Performance evaluation of D-shaped photonic crystal fiber based SPR sensors with different plasmonic materials: a comparative analysis. Results Eng. 26, 104715. 10.1016/j.rineng.2025.104715

[B7] El-assar M. Taha T. E. El-Samie F. E. A. Fayed H. A. Aly M. H. (2023). Zinc selenide based dual-channel SPR optical biosensor for HIV genome DNA hybridization detection. Opt. Quantum Electron. 55 (13), 1143. 10.1007/s11082-023-05296-5

[B8] Englebienne P. Hoonacker A. V. Verhas M. (2003). Surface plasmon resonance: principles, methods and applications in biomedical sciences. J. Spectrosc. 17 (2-3), 255–273. 10.1155/2003/372913

[B9] Fan Q. Tang G. Jiang M. Xu Y. Pan N. Liang Z. (2025). Clinical prognostic value of TTV and HCMV but not EBV for outcomes in hospitalized HIV-Positive patients. Biosaf. Health 7, 173–182. 10.1016/j.bsheal.2025.05.006 40693040 PMC12276542

[B10] Hossain M. B. Kabir M. A. Hossain M. S. Islam K. Z. Hossain M. S. Pathan M. I. (2020). Numerical modeling of MoS2–Graphene bilayer-based high-performance surface plasmon resonance sensor: structure optimization for DNA hybridization. Opt. Eng. 59, 105105. 10.1117/1.oe.59.10.105105

[B11] Hu S. M. Gregor L. V. (1967). Silicon nitride films by reactive sputtering. J. Electrochem. Soc. 114 (8), 826. 10.1149/1.2426749

[B12] Huang F. Xie Z. Zhang Q. Zada S. Lin R. Deng Y. (2025). Recent advances in fluorescence resonance energy transfer (FRET) biosensors for exosomes. Curr. Issues Mol. Biol. 47 (4), 235. 10.3390/cimb47040235 40699635 PMC12025574

[B13] Jain S. Trivedi R. Banshiwal J. K. Singh A. S. Chakraborty B. (2023). “Two-dimensional materials (2DMs): classification, preparations, functionalization and fabrication of 2DMs-oriented electrochemical sensors,” in 2D materials-based electrochemical sensors (Elsevier), 45–132.

[B14] Kapaklis V. Poulopoulos P. Karoutsos V. Manouras T. Politis C. (2006). Growth of thin Ag films produced by radio frequency magnetron sputtering. Thin Solid Films 510 (1-2), 138–142. 10.1016/j.tsf.2005.12.311

[B15] Karouta F. Vora K. Tian J. Jagadish C. (2012). Structural, compositional and optical properties of PECVD silicon nitride layers. J. Phys. D Appl. Phys. 45 (44), 445301. 10.1088/0022-3727/45/44/445301

[B16] Kukanskis K. Elkind J. Melendez J. Murphy T. Miller G. Garner H. (1999). Detection of DNA hybridization using the TISPR-1 surface plasmon resonance biosensor. Anal. Biochem. 274 (1), 7–17. 10.1006/abio.1999.4241 10527491

[B17] Kumar A. Kumar A. Srivastava S. K. (2022). Silicon nitride-BP-based surface plasmon resonance highly sensitive biosensor for virus SARS-CoV-2 detection. Plasmonics 17 (3), 1065–1077. 10.1007/s11468-021-01589-1 35103050 PMC8791766

[B18] Kumar A. Yadav A. K. Kushwaha A. S. Srivastava S. K. (2020). A comparative study among WS2, MoS2 and graphene based surface plasmon resonance (SPR) sensor. Sensors Actuators Rep. 2 (1), 100015. 10.1016/j.snr.2020.100015

[B19] Lin L. L. Alvarez-Puebla R. Liz-Marzán L. M. Trau M. Wang J. Fabris L. (2025). Surface-enhanced raman spectroscopy for biomedical applications: recent advances and future challenges. ACS Appl. Mater. and Interfaces 17 (11), 16287–16379. 10.1021/acsami.4c17502 39991932 PMC12184206

[B20] Liu Z. Murphy A. W. A. Kuppe C. Hooper D. C. Valev V. K. Ilie A. (2019). WS2 nanotubes, 2D nanomeshes, and 2D in-plane films through one single chemical vapor deposition route. ACS nano 13 (4), 3896–3909. 10.1021/acsnano.8b06515 30912636 PMC7007277

[B21] Martiskainen I. Juntunen E. Salminen T. Vuorenpää K. Bayoumy S. Vuorinen T. (2021). Double-antigen lateral flow immunoassay for the detection of anti-HIV-1 and-2 antibodies using upconverting nanoparticle reporters. Sensors 21 (2), 330. 10.3390/s21020330 33418986 PMC7825344

[B22] Miczi M. Diós Á. Bozóki B. Tőzsér J. Mótyán J. A. (2021). Development of a bio-layer interferometry-based protease assay using HIV-1 protease as a model. Viruses 13 (6), 1183. 10.3390/v13061183 34205716 PMC8235736

[B23] Nurrohman D. T. Chiu N. F. (2020). Surface plasmon resonance biosensor performance analysis on 2D material based on graphene and transition metal dichalcogenides. ECS J. Solid State Sci. Technol. 9, 115023. 10.1149/2162-8777/abb419

[B24] Pandey P. S. Kumar Raghuwanshi S. Kumar S. (2021). Recent advances in two-dimensional materials-based kretschmann configuration for SPR sensors: a review. IEEE Sensors J. 22 (2), 1069–1080. 10.1109/jsen.2021.3133007

[B25] Pravesh R. Kumar D. Pandey B. P. Chaudhary V. S. Kumar S. (2024). Design and analysis of a double D-shaped dual core PCF sensor for detecting biomolecules in the human body. IEEE Sensors J. 24, 14159–14166. 10.1109/jsen.2024.3380095

[B26] Rahman M. S. Anower M. S. Hasan M. R. Hossain M. B. Haque M. I. (2017). Design and numerical analysis of highly sensitive Au–MoS2-graphene-based hybrid surface plasmon resonance biosensor. Opt. Commun. 396, 36–43. 10.1016/j.optcom.2017.03.035

[B27] Sang M. Kim K. Lee D. J. Cho Y. U. Lee J. W. Yu K. J. (2025). Technical roadmap of ultra-thin crystalline silicon-based bioelectronics. Int. J. Extreme Manuf. 7, 052006. 10.1088/2631-7990/add7a4

[B28] Satija N. Patel F. Schmidt G. Doanman D. V. Kapoor M. La Porte A. (2025). Tracking HIV persistence across T cell lineages during early ART-Treated HIV-1-infection using a reservoir-marking humanized mouse model. Nat. Commun. 16 (1), 2233. 10.1038/s41467-025-57368-7 40044684 PMC11883074

[B29] Sawraj S. Kumar D. Pravesh R. Chaudhary V. S. Pandey B. P. Sharma S. (2024). PCF-Based sensors for biomedical Applications-A review. IEEE Trans. nanobioscience 24, 157–164. 10.1109/tnb.2024.3462748 39288060

[B30] Shi Q. Feng S. Zhao J. (2025). Engineering design of an expandable 1-D photonic crystal slab biosensor array for joint detection of multiple tumor markers. IEEE Sensors J. 25, 5986–5994. 10.1109/jsen.2024.3523479

[B31] Singh S. Kumar D. Sahu A. Chaudhary V. S. Singh G. Kumar S. (2025). Photonic crystal fiber based sensors for various cancer detection in human Body-A review. IEEE Sensors J. 25, 5956–5968. 10.1109/jsen.2024.3524325

[B32] Ten Brink D. Martin-Hughes R. Bowring A. L. Wulan N. Burke K. Tidhar T. (2025). Impact of an international HIV funding crisis on HIV infections and mortality in low-income and middle-income countries: a modelling study. Lancet HIV 12 (5), e346–e354. 10.1016/s2352-3018(25)00074-8 40157378

[B33] Tene T. Arias F. A. Guamán-Lozada D. F. Guadalupe Alcoser M. A. Gahramanli L. Vacacela Gomez C. (2025d). Advanced SPR-based biosensors for potential use in cancer detection: a theoretical approach. Sensors 25 (9), 2685. 10.3390/s25092685 40363123 PMC12074272

[B34] Tene T. Arias Arias F. Paredes-Páliz K. I. Cunachi Pillajo A. M. Flores Huilcapi A. G. Carrera Almendariz L. S. (2025b). WS2/Si3N4-Based biosensor for low-concentration coronavirus detection. Micromachines 16 (2), 128. 10.3390/mi16020128 40047597 PMC11857482

[B35] Tene T. Coello-Fiallos D. Borja M. Sánchez N. Londo F. Gomez C. V. (2025a). Surface plasmon resonance biosensors for SARS-CoV-2 sensing: the role of silicon nitride and graphene. Biosens. Bioelectron. X 23, 100586. 10.1016/j.biosx.2025.100586

[B36] Tene T. Coello-Fiallos D. Robalino M. D. L. P. Londo F. Gomez C. V. (2025e). The effect of MoS2 and Si3N4 in surface plasmon resonance biosensors for HIV DNA hybridization detection: a numerical study. Micromachines 16 (3), 295. 10.3390/mi16030295 40141905 PMC11946481

[B37] Tene T. Guevara M. Romero P. Guapi A. Gahramanli L. Vacacela Gomez C. (2024). SARS-CoV-2 detection by surface plasmon resonance biosensors based on graphene-multilayer structures. Front. Phys. 12, 1503400. 10.3389/fphy.2024.1503400

[B38] Tene T. Vique López D. F. Valverde Aguirre P. E. Monge Moreno A. M. Vacacela Gomez C. (2025c). The detection of different cancer types using an optimized MoS2-Based surface plasmon resonance multilayer system. Sci 7 (2), 76. 10.3390/sci7020076

[B39] Wong Z. W. New S. Y. (2025). Recent advances in biosensors based on hybridization chain reaction and silver nanoclusters. Small Methods 9, 2401436. 10.1002/smtd.202401436 39757735

[B40] Wu L. Chu H. S. Koh W. S. Li E. P. (2010). Highly sensitive graphene biosensors based on surface plasmon resonance. Opt. express 18 (14), 14395–14400. 10.1364/oe.18.014395 20639924

[B41] Yan J. Lian S. Cao Z. Du Y. Wu P. Sun H. (2023). CVD controlled preparation and growth mechanism of 2H-WS2 nanosheets. Vacuum 207, 111564. 10.1016/j.vacuum.2022.111564

[B42] Yorulmaz B. Özden A. Şar H. Ay F. Sevik C. Perkgöz N. K. (2019). CVD growth of monolayer WS2 through controlled seed formation and vapor density. Mater. Sci. Semicond. Process. 93, 158–163. 10.1016/j.mssp.2018.12.035

[B43] Zhang X. Wu S. Lin Y. Zhang W. Zhang Y. Li X. (2025a). Development of an assay evaluating the inducible HIV‐1 latent reservoir based on reverse transcription droplet digital PCR for unspliced/intact viral RNA. J. Med. Virology 97 (3), e70295. 10.1002/jmv.70295 40088087

[B44] Zhang X. Zhang F. Ren J. Wang Y. Li Z. Li X. (2025b). Excitation-detection integrated trapezoidal prism-coupled surface plasmon resonance based on a compact optical system. Sensors Actuators B Chem. 427, 137234. 10.1016/j.snb.2025.137234

