# Supplementary material for: Numerical analysis of WS2/Si3N4 for improved SPR-based HIV DNA detection
Source: Front Bioeng Biotechnol. 2025 Jul 17;13:1577925. doi: 10.3389/fbioe.2025.1577925 (PMC12310735; doi:10.3389/fbioe.2025.1577925)
Supplement: Supplementary file 1 [file DataSheet1.docx]

**SUPPLEMENTARY INFORMATION: Numerical Analysis of WS_2_/Si_3_N_4_ for Improved SPR-Based HIV DNA Detection**

Talia Tene^1,^*, Yesenia Cevallos^2,3^, Jessica Alexandra Marcatoma Tixi^4^, Natalia Alexandra Pérez Londo^4^, Lala Gahramanli^5,6^, Cristian Vacacela Gomez^7,^*

^1^ Department of Chemistry, Universidad Técnica Particular de Loja, Loja 110160, Ecuador

^2^ College of Engineering, Universidad Nacional de Chimborazo, Riobamba 060108, Ecuador

^3^ Universidad San Francisco de Quito IMNE, Diego de Robles s/n, Cumbayá, Quito 170901, Ecuador

^4^ Carrera de Estadística, Facultad de Ciencias, Escuela Superior Politécnica de Chimborazo (ESPOCH), Riobamba 060155, Ecuador

^5^ Nano Research Laboratory, Excellent Center, Baku State University, Baku, Azerbaijan

^6^ Chemical Physics of Nanomaterials, Physics Department, Baku State University, Baku, Azerbaijan

^7^ INFN-Laboratori Nazionali di Frascati, Via E. Fermi 54, 00044 Frascati, Italy

Correspondence: [tbtene@utpl.edu.ec](mailto:tbtene@utpl.edu.ec) (T.T.) & [cristianisaac.vacacelagomez@utpl.edu.ec](mailto:cristianisaac.vacacelagomez@utpl.edu.ec) (C.V.G.)

**Supplementary Figures**

**
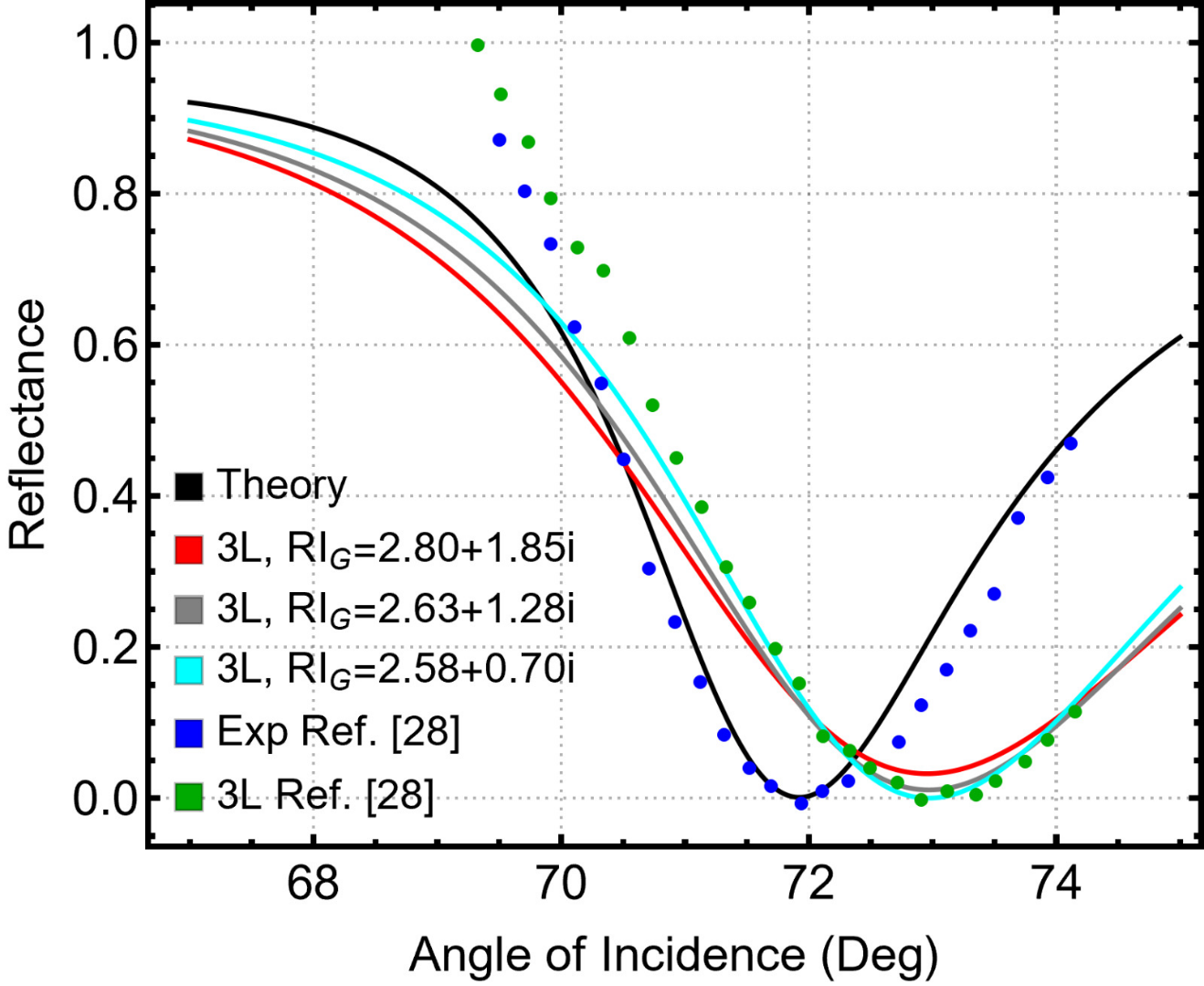
**

**Figure S1.** Theoretical model predictions versus the experimental data reported in Ref. [28].

**
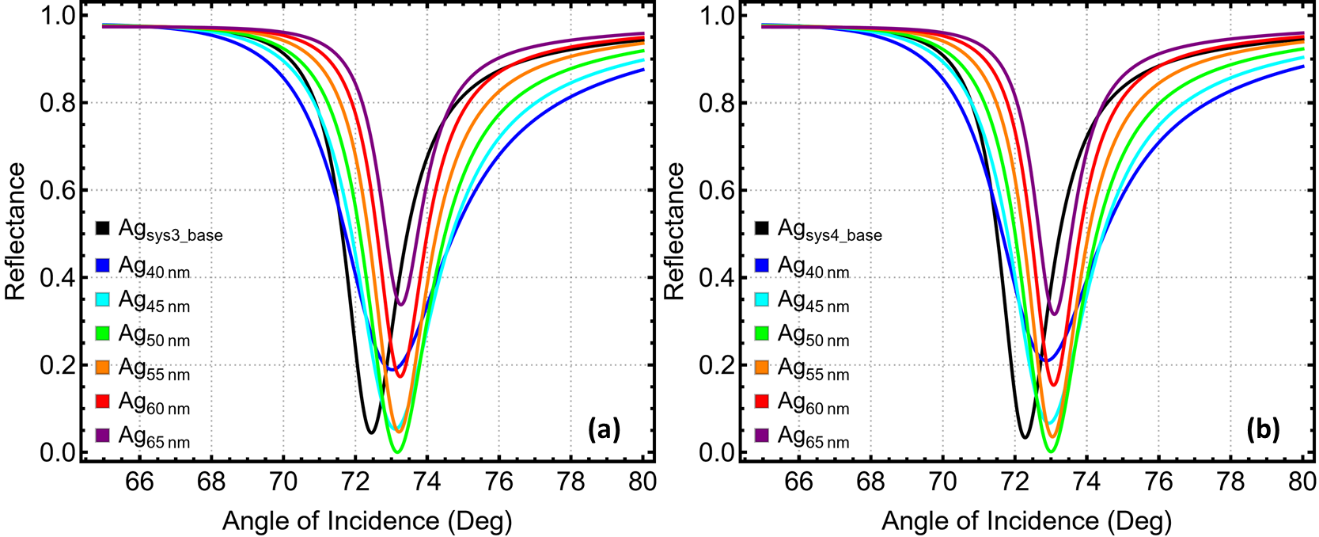
**

**Figure S2.** Reflectance profiles as a function of the silver-thickness series. (a) Sys₃ baseline (black) compared with variants containing 40, 45, 50, 55, 60, and 65 nm Ag films. (b) Corresponding data for Sys₄.

**
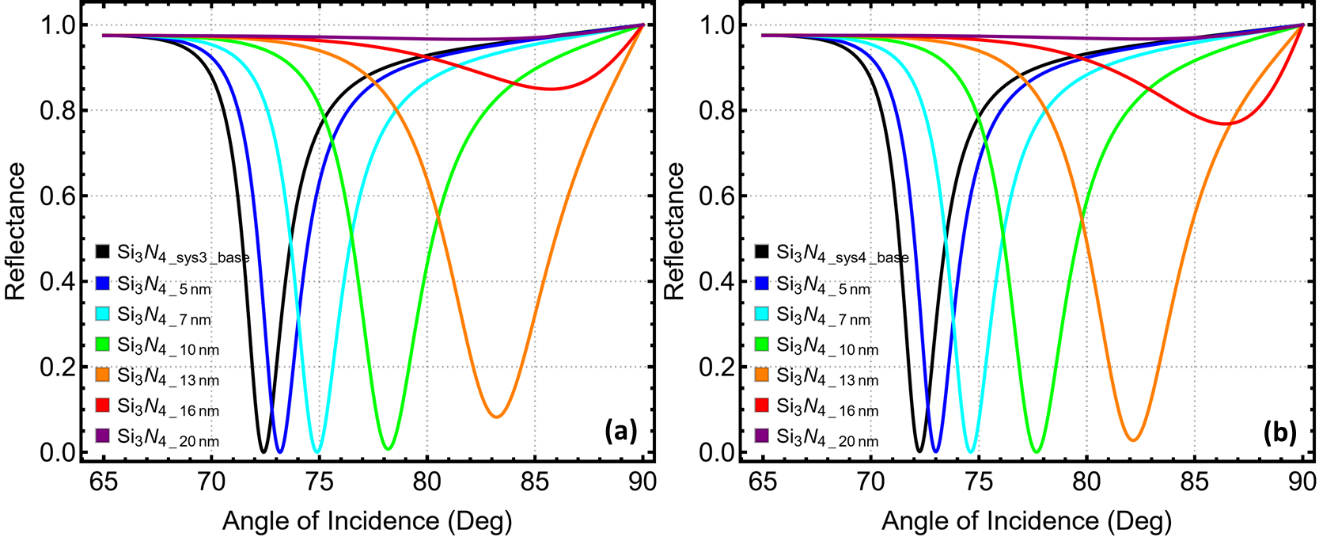
**

**Figure S3.** Reflectance profiles as a function of the Si₃N₄-thickness series. (a) Sys₃ baseline (black) compared with variants containing 5, 7, 10, 13, 16, and 20 nm Si₃N₄ spacers. (b) Corresponding data for Sys₄.

**
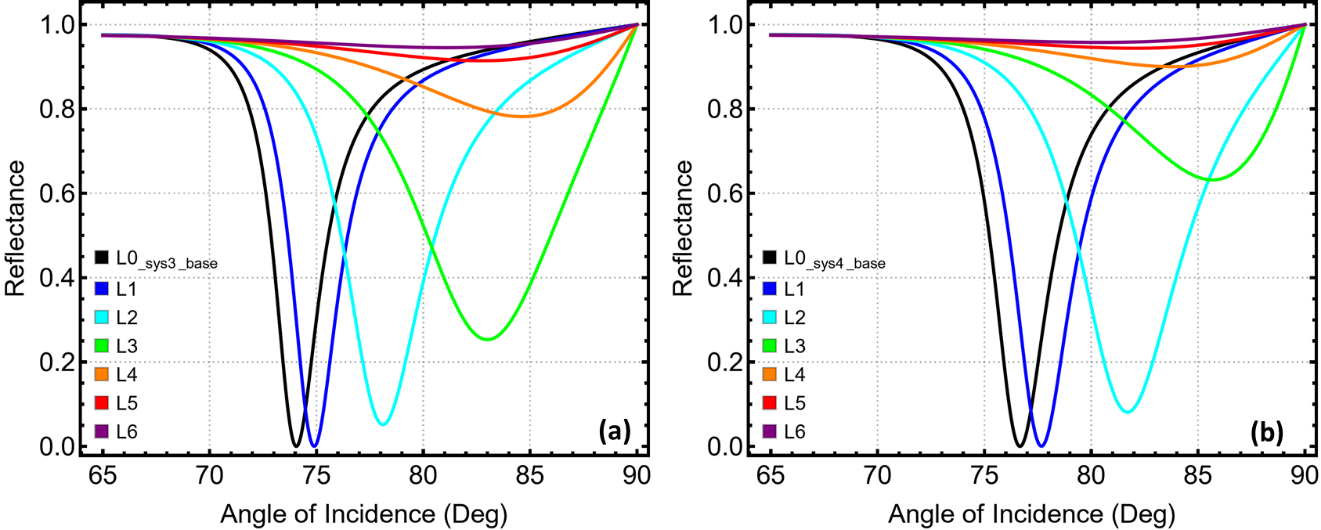
**

**Figure S4.** Reflectance profiles as a function of the WS₂-layer series. (a) Sys₃ baseline with one layer without HIV DNA hybridisation (black) compared with one to six WS₂ layers (L1–L6). (b) Corresponding data for Sys₄.

**Supplementary Tables**

**Table S1.** Codes and nomenclature for the five simulated configurations, showing system number, layer sequence, and the shorthand label adopted in figures and text.

| **Sys No.** | **Code** | **Full Name** | **Nick Name** |
| --- | --- | --- | --- |
| 0 | Sys_0_ | Prism/Silver/PBS Medium | P/Ag/M_PBS_ |
| 1 | Sys_1_ | Prism/Silver/PBS+HIV Medium | P/Ag/M_PBS+HIV_ |
| 2 | Sys_2_ | Prism/Silver/Si_3_N_4_/ PBS+HIV Medium | P/Ag/SiN/M_PBS+HIV_ |
| 3 | Sys_3_ | Prism/Silver/Si_3_N_4_/Tungsten Disulfide/PBS+HIV Medium | P/Ag/SiN/WS_2_/M_PBS+HIV_ |
| 4 | Sys_4_ | Prism/Silver/Tungsten Disulfide/Si_3_N_4_/PBS+HIV Medium | P/Ag/WS_2_/SiN/M_PBS+HIV_ |

**Table S2.** Refractive indices and nominal thicknesses employed in the transfer-matrix calculations. Metal values are listed as n + ik at 633 nm; substrate and fluidic media are treated as semi-infinite.

| **Material** | **Refractive Index** | **Thickness (nm)** | **Ref.** |
| --- | --- | --- | --- |
| BK-7 (P) | 1.5151 | --- | [30] |
| Silver (Ag) | 0.056253 + 4.2760 i | 55.0 | [31] |
| Si_3_N_4_ (SiN) | 2.0394 | 5.00 | [31] |
| Tungsten Disulfide (WS_2_) | 4.9 + 0.3124 i | 0.80 | [32] |
| PBS (M) | 1.335 | --- | [30] |
| PBS + DNA hybridization (BSA+Strep.+dsDNA) | 1.340 | --- | [33] |

**Table S3.** Surface‐plasmon parameters extracted from the reflectance curves for Sys₁–Sys₄, listing resonance angle, minimum reflectance expressed as attenuation, full width at half‐minimum, and percentage increase in angular sensitivity relative to the baseline (Sys_0_).

| **Sys No.** | **Code** | **SPR Peak position** | **Attenuation (%)** | **FWHM (nm)** | **Sensitivity Enhancement (%)** |
| --- | --- | --- | --- | --- | --- |
| 1 | Sys_1_ | 68.65 | 0.02 | 0.93 | 0.86 |
| 2 | Sys_2_ | 71.29 | 0.01 | 1.29 | 4.74 |
| 3 | Sys_3_ | 73.21 | 4.65 | 2.09 | 7.57 |
| 4 | Sys_4_ | 73.04 | 3.53 | 1.99 | 7.32 |

**Table S4.** Simulated SPR parameters for Sys₃ and Sys₄ as a function of silver thickness (40–65 nm). Columns list resonance angle, minimum‐dip attenuation, full width at half‐minimum (FWHM), and percentage sensitivity increase relative to the 55 nm film for each system.

| **Thickness (nm)** | **SPR Peak position** | **Attenuation (%)** | **FWHM (nm)** | **Sensitivity Enhancement (%)** |
| --- | --- | --- | --- | --- |
| **Sys_3_** | | | | |
| 40 | 73.03 | 18.87 | 3.93 | 0.80 |
| 45 | 73.11 | 5.31 | 3.06 | 0.91 |
| 50 | 73.17 | 0.05 | 2.47 | 0.99 |
| 55 | 73.21 | 4.65 | 2.09 | 1.06 |
| 60 | 73.25 | 17.31 | 1.86 | 1.10 |
| 65 | 73.27 | 33.76 | 1.75 | 1.13 |
| **Sys_4_** | | | | |
| 40 | 72.88 | 20.97 | 3.84 | 0.82 |
| 45 | 72.95 | 6.65 | 2.97 | 0.91 |
| 50 | 73.01 | 0.12 | 2.38 | 0.99 |
| 55 | 73.05 | 3.53 | 1.99 | 1.05 |
| 60 | 73.08 | 15.37 | 1.76 | 1.09 |
| 65 | 73.10 | 31.57 | 1.64 | 1.12 |

**Table S5.** Simulated SPR parameters for Sys₃ and Sys₄ as a function of Si₃N₄ spacer thickness (5–20 nm). Columns list resonance angle, minimum‐dip attenuation, full width at half‐minimum (FWHM), and percentage sensitivity increase relative to the 5 nm spacer for each system.

| **Thickness (nm)** | **SPR Peak position** | **Attenuation (%)** | **FWHM (nm)** | **Sensitivity Enhancement (%)** |
| --- | --- | --- | --- | --- |
| **Sys_3_** | | | | |
| 5 | 73.16 | 0.01 | 2.51 | 1.03 |
| 7 | 74.89 | 0.03 | 2.93 | 3.41 |
| 10 | 78.19 | 0.76 | 3.76 | 7.97 |
| 13 | 83.21 | 8.22 | 5.11 | 14.91 |
| 15 | 85.72 | 84.94 | 10.24 | 18.37 |
| 20 | 81.85 | 96.60 | 133.60 | 13.03 |
| **Sys_4_** | | | | |
| 5 | 73.00 | 0.12 | 2.41 | 1.03 |
| 7 | 74.61 | 0.01 | 2.77 | 3.27 |
| 10 | 77.66 | 0.05 | 3.46 | 7.49 |
| 13 | 82.14 | 2.77 | 4.53 | 13.69 |
| 15 | 86.40 | 76.81 | 8.07 | 19.59 |
| 20 | 82.50 | 96.69 | 186.41 | 14.18 |

**Table S6.** Simulated SPR parameters for Sys₃ and Sys₄ as a function of WS₂ layer count (L1–L6). Columns list resonance angle, minimum‐dip attenuation, full width at half‐minimum (FWHM), and percentage sensitivity increase relative to the single‐layer case without HIV DNA hybridisation for each system.

| **Layers** | **SPR Peak position** | **Attenuation (%)** | **FWHM (nm)** | **Sensitivity Enhancement (%)** |
| --- | --- | --- | --- | --- |
| **Sys_3_** | | | | |
| L1 | 74.89 | 0.03 | 2.93 | 1.12 |
| L2 | 78.10 | 5.16 | 4.57 | 5.46 |
| L3 | 83.00 | 25.29 | 6.97 | 12.07 |
| L4 | 84.61 | 78.16 | 10.68 | 14.25 |
| L5 | 82.75 | 91.41 | 15.84 | 11.73 |
| L6 | 81.04 | 94.50 | 19.72 | 9.42 |
| **Sys_4_** | | | | |
| L1 | 77.66 | 0.05 | 3.46 | 1.30 |
| L2 | 81.69 | 8.11 | 5.31 | 6.55 |
| L3 | 85.67 | 63.16 | 8.32 | 11.75 |
| L4 | 83.82 | 89.99 | 13.94 | 9.33 |
| L5 | 82.01 | 94.37 | 19.20 | 6.97 |
| L6 | 80.41 | 95.76 | 34.57 | 4.89 |

**Table S7.** Optimized parameters of Sys_2_ and Sys_3_ configurations

| **Material** | **Refractive Index (RI)** | **Thickness (nm)** |
| --- | --- | --- |
| **Sys_3_** | | |
| BK7 (P) | 1.5151 | --- |
| Ag | 0.056253 + 4.2760 | 50.0 |
| Si_3_N_4_ (SiN) | 2.0394 | 7.0 |
| Tungsten Disulfide (WS_2_) | 4.9 + 0.3124 i | 0.8*L (L=1) |
| PBS (M) | 1.335 |  |
| **Sys_4_** | | |
| BK7 (P) | 1.5151 | --- |
| Ag | 0.056253 + 4.2760 | 50.0 |
| Si_3_N_4_ (SiN) | 2.0394 | 10.0 |
| Tungsten Disulfide (WS_2_) | 4.9 + 0.3124 i | 0.8*L (L=1) |
| PBS (M) | 1.335 | --- |
| **HIV in PBS solution** | | |
| HIV genome DNA (BSA + Strep. + dsDNA)@25 ^o^C | 1.340 | --- |

**Table S8.** Comparison of SPR characteristics for Sys₃ and Sys₄ under baseline (PBS) and hybridised (PBS + HIV) conditions. Columns list resonance angle, minimum reflectance expressed as attenuation, full width at half-minimum (FWHM), and the resulting sensitivity enhancement relative to the corresponding PBS state. RIU is refractive index unit

| **Configuration** | **SPR Peak position** | **Attenuation %** | **FWHM (nm)** | **Sensitivity Enhancement (%)** |
| --- | --- | --- | --- | --- |
| **Sys_3-PBS_** | 74.05 | 0.04 | 2.82 | 0.0 |
| **Sys_3-PBS+HIV_** | 74.89 | 0.04 | 2.93 | 1.12 |
| **Sys_4-PBS_** | 76.66 | 0.06 | 3.31 | 0.0 |
| **Sys_4-PBS+HIV_** | 77.67 | 0.06 | 3.46 | 1.31 |

**Table S9.** Key performance metrics for the two optimised configurations under HIV‐DNA hybridisation. Columns list angular shift (Δθ), refractive‐index sensitivity (S), detection accuracy (DA), and quality factor (QF). RIU is refractive index unit

| **Configuration** | $\boldsymbol{\Delta\theta}$ | ***S* (**$\boldsymbol{^{\circ}/RIU}$**)** | **DA** | **QF (*RIU*^-1^)** |
| --- | --- | --- | --- | --- |
| **Sys_3-PBS+HIV_** | 0.83 | 167 | 0.28 | 56.89 |
| **Sys_4-PBS+HIV_** | 1.01 | 201 | 0.29 | 57.98 |

**Table S10.** Figure of merit (FoM), estimated limit of detection (LoD, 10⁻⁵ RIU), and Comprehensive Sensitivity Factor (CSF) for Sys₃ and Sys₄ under HIV-DNA hybridisation. RIU is refractive index unit

| **Configuration** | **FoM (*RIU*^-1^)** | **LoD (10^-5^)** | **CSF** |
| --- | --- | --- | --- |
| **Sys_3-PBS+HIV_** | 56.86 | 2.99 | 55.48 |
| **Sys_4-PBS+HIV_** | 57.95 | 2.48 | 56.55 |
